# *Shigella* Effector VirA Suppresses Plant Immunity by Compromising PRA1.F3‐Dependent Accumulation of FLS2 at the Plasma Membrane

**DOI:** 10.1111/pce.70541

**Published:** 2026-04-14

**Authors:** Sung Hee Jo, Sukyoung Jung, Myoung Hui Lee, Inhwan Hwang, Jeong Mee Park

**Affiliations:** ^1^ Molecular Plant Immunity Research Laboratory, Plant Systems Engineering Research Center, Korea Research Institute of Bioscience & Biotechnology (KRIBB) Daejeon South Korea; ^2^ Department of Life Sciences Graduate School of Korea University Seoul South Korea; ^3^ Department of Life Sciences Pohang University of Science and Technology Pohang South Korea; ^4^ Current Address: Wheat Research Team, National Institute of Crop Science Rural Development Administration Wanju South Korea

**Keywords:** endomembrane regulation, FLS2, plant immunity, PRA1 isoforms, *Shigella*, T3SS, VirA

## Abstract

Surface immune receptors such as flagellin‐sensitive 2 (FLS2) are critical for plant defence, and their accumulation at the plasma membrane is tightly controlled by the endomembrane system. Here, we identify the *Arabidopsis thaliana* prenylated Rab acceptor PRA1.F3 as a positive regulator required for efficient accumulation of FLS2 at the plasma membrane and as a key target of the *Shigella* type III effector VirA. Although VirA can associate with multiple PRA1 isoforms, immune suppression and enhanced pathogen proliferation in *Arabidopsis* specifically depend on PRA1.F3. VirA associates with PRA1.F3 at endomembrane compartments and promotes its ubiquitin‐proteasome‐dependent degradation, leading to reduced FLS2 abundance and attenuation of pattern‐triggered immunity. This immune‐suppressive activity requires both the GTPase‐activating function and membrane association of VirA. In contrast, the closely related isoform PRA1.F4 negatively affects FLS2 accumulation but does not undergo VirA‐dependent destabilization. Together, these findings establish PRA1.F3 as a central regulator of immune receptor accumulation and reveal a selective bacterial strategy to subvert host endomembrane regulation and evade immune recognition.

## Summary statement

- The *Shigella* type III effector VirA promotes ubiquitin‐proteasome‐dependent degradation of *Arabidopsis* PRA1.F3, thereby reducing plasma membrane accumulation of the immune receptor FLS2 and suppressing pattern‐triggered immunity.

## 1 Introduction

Plants rely on two interconnected layers of innate immunity to defend against pathogens (Jones and Dangl 2006; Boller and Felix 2009). The first layer, known as pattern‐triggered immunity (PTI), is initiated at the plasma membrane (PM) by pattern recognition receptors (PRRs) that detect conserved pathogen‐associated molecular patterns. To overcome PTI, pathogens inject effector proteins into host cells via the type III secretion system (T3SS) in an attempt to disrupt immune components. In turn, plants deploy a second layer of immunity, known as effector‐triggered immunity (ETI), which is mediated by intracellular nucleotide‐binding leucine‐rich repeat (NB‐LRR) proteins that recognise these effectors. Despite distinct recognition mechanisms and subcellular localisations, PTI and ETI converge on shared downstream defence responses (Thomma et al. 2011; Dodds et al. 2024). Recent studies suggest that ETI can potentiate PTI by activating PRR pathways, highlighting their mutual reinforcement (Thomma et al. 2011; Ngou et al. 2021; Yuan et al. 2021).

Intracellular trafficking via the endomembrane system is essential for the precise establishment of both PTI and ETI (Gu et al. 2017). In plants, newly synthesised PRRs are transported from the endoplasmic reticulum (ER) to the PM via the conventional secretory pathway. Upon ligand binding, activated PRRs are internalised through endocytosis, sorted into endosomes for either recycling back to the PM or degradation in the vacuole (Robatzek et al. 2006; Mbengue et al. 2016). Unlike animal PRRs, which are distributed across various organelles, plant PRRs are predominantly PM‐localised. During coevolution with hosts, many pathogens have evolved strategies to interfere with PRR trafficking as a means of immune evasion. Notably, plants may counteract these strategies by rerouting defence proteins through unconventional secretory pathways, such as ER bodies or autophagy, bypassing the Golgi (Yuen et al. 2023). It remains unclear whether and how pathogen effectors selectively exploit such trafficking rewiring during immune suppression, underscoring the need to understand how PRR trafficking is regulated during immune activation.

Effector‐targeted trafficking components have provided valuable insights into plant immunity. Several pathogen effectors target Rab GTPases such as Rab8 and Rab11, which are involved in PR1 secretion and flagellin‐sensitive 2 (FLS2) trafficking (Speth et al. 2009; Yuen et al. 2023). For instance, the *Pseudomonas syringae* effector AvrPto was reported to interact with Rab8, although this has not been validated in planta. Similarly, AVR3a from *Phytophthora infestans* impairs internalisation of activated FLS2, but its impact on signalling remains ambiguous (Chaparro‐Garcia et al. 2015). Other effectors such as HopM1 degrade the trans‐Golgi network (TGN)‐localised protein MIN7 via the host proteasome (Nomura et al. 2011), while AvrPtoB and XopP inhibit EXO70 subunits of the exocyst complex to suppress defence‐related secretion (Wang et al. 2019; Michalopoulou et al. 2022). Despite these advances, the effector‐mediated manipulation of specific trafficking regulators that directly control immune receptor homoeostasis remains poorly understood, in part because membrane trafficking is highly dynamic and implicated in multiple plant physiological processes beyond immunity.

A common effector strategy is targeting Rab GTPases, which regulate vesicle fusion and cargo specificity (Rivero et al. 2019). Rabs are post‐translationally modified by prenylation and maintained in the cytosol by GDP dissociation inhibitors (Molendijk et al. 2004). Prenylated Rab acceptor 1 (PRA1) proteins, which were originally identified as Rab‐binding proteins, interact with a broad range of prenylated and non‐prenylated partners, including viral proteins (Huang et al. 2001; Li et al. 2001; Calero and Collins 2002; Abu Irqeba and Ogilvie 2019). In *Arabidopsis thaliana*, 19 PRA1 isoforms are differentially localised within the endomembrane system (Alvim Kamei et al. 2008), and some, such as PRA1.F4 and PRA1.B6, have been implicated in vesicle trafficking, protein sorting, and stress responses (Lee et al. 2011; Lee et al. 2017). Moreover, a recent study reported that PRA1 plays a functional role in NLR‐mediated immunity in rice (Liang et al. 2025), suggesting this family has broader relevance in plant immune signalling.

*Shigella* spp. are Gram‐negative enteric pathogens primarily adapted to human hosts and are transmitted via contaminated food or water (Pakbin et al. 2021). Remarkably, *Shigella* can also colonise plants and proliferates in the apoplast using its T3SS (Jo et al. 2019). Several *Shigella* effectors, such as OspF, which suppresses mitogen‐activated protein kinase (MAPK) activation, function as virulence factors in both animal and plant systems, highlighting the conservation of effector activities across kingdoms (Jo et al. 2019). Although *Shigella* strains differ in their ability to proliferate in plants, they do not appear to invade plant cells as they do epithelial cells in mammals.

Among *Shigella* T3SS effectors, VirA was originally identified as a key virulence factor required for bacterial invasion and intercellular spread in mammalian epithelial cells (Uchiya et al. 1995). Early sequence‐based analyses suggested that VirA might function as a cysteine protease, and mutation of a conserved cysteine residue at position 34 (C34S) was shown to abolish VirA‐dependent virulence phenotypes in animal infection models, including defects in membrane ruffling and intracellular dissemination (Yoshida et al. 2006). However, subsequent biochemical studies demonstrated that VirA lacks detectable cysteine protease activity in vitro, indicating that the virulence defect associated with the C34S mutation cannot be attributed to loss of protease activity (Germane et al. 2008). Later structural and functional analyses revealed that VirA acts as a GTPase‐activating protein (GAP) for Rab1, thereby interfering with ER‐to‐Golgi trafficking in mammalian cells (Dong et al. 2012). Despite these advances, the molecular role of the conserved C34 residue in VirA function remains unresolved, as it is unclear whether C34 influences VirA activity through effects on protein stability, subcellular localisation, or interactions with host cellular factors rather than enzymatic function. Furthermore, whether VirA‐mediated disruption of host trafficking pathways contributes to its virulence in plant hosts has yet to be determined.

In this study, we identify PRA1.F3, a previously uncharacterised isoform of the *Arabidopsis* PRA1 family, as a biologically relevant target of the *Shigella* T3SS effector VirA. While initial yeast two‐hybrid (Y2H) screening identified PRA1.E as a candidate interactor, subsequent functional analyses in *Arabidopsis* demonstrated that PRA1.F3, but not other PRA1 isoforms, mediates VirA‐dependent immune suppression. We demonstrate that VirA facilitates PRA1.F3 degradation via the ubiquitin‐proteasome system, leading to reduced FLS2 accumulation at the PM and suppression of pattern‐triggered immunity. These findings uncover an essential immune‐regulatory role for PRA1.F3 and reveal a conserved bacterial strategy that subverts host vesicle trafficking to promote virulence.

## 2 Materials and Methods

### 2.1 Plant Materials and Growth Conditions

*Arabidopsis thaliana* ecotype Col*‐*0 was used as the WT in this study. Col‐0 and five *Arabidopsis* mutants, namely, *pra1.b5* (SALK_043145), *pra1.e* (SAIL_1307_G02), *pra1.f3* (SALK_079876), *pra1.f4* (GABI‐114H12), and *pra1.g1* (GABI_292C11), were obtained from the Arabidopsis Biological Resource Center (Columbus, OH, USA) (Table S1). Homozygotes of these mutants were screened and confirmed by genomic DNA‐based PCR. Arabidopsis and *N. benthamiana* plants were grown at 22 ± 2°C under a 16 h light/8 h dark cycle in plastic pots containing steam‐sterilised mixed soil (2:1:1 = soil:vermiculite:perlite, v/v/v) (Moon et al. 2016). Seeds were sterilised using 70% (v/v) ethanol for 2 min and 50% household bleach with gentle shaking for 5 min, and then rinsed with sterile deionized water. The sterilised seeds were sown on half‐strength Murashige and Skoog (1/2 MS) medium‐ and 0.6% (w/v) agar‐containing plates (Duchefa) supplemented with 1% sucrose (Murashige and Skoog 1962).

### 2.2 Bacterial Strains and Growth Conditions

*S. flexneri* 5a was grown at 37°C in Luria‐Bertani (LB) medium or tryptic soy broth (Sigma‐Aldrich). *Pseudomonas syringae* pv. tomato DC3000 (*Pst*) was grown in King's B medium (KisanBio) supplemented with appropriate antibiotics (King et al. 1954; Runyen‐Janecky and Payne 2002) at 28°C with shaking at 200 rpm.

ΔvirA was constructed by Dr. Ryu and colleagues using the lambda red recombination method, as described previously (Lee et al. 2018). To generate complementation lines, the coding DNA sequence (CDS) of the WT *VirA* gene was cloned into the pUC18 vector harbouring the ampicillin marker, and the resulting construct was transformed into the ΔvirA mutant.

### 2.3 Plasmid Constructs

The plasmids and PCR primers used in this study are listed in Tables S1 and S2. To perform Gateway cloning, the coding sequences of *VirA* and *PRA1* isoforms were PCR‐amplified, and PCR products were cloned into the pDONR™207 vector by BP recombination using Gateway BP Clonase™ II enzyme mix (Invitrogen). The resulting constructs were subcloned into the pBAV178, pBAV179, pK7FWG2, pGWB11, pGWB454/455, and pk7YWG2/pk7WGY2 vectors by LR recombination (Gateway LR Clonase II, Invitrogen).

To generate HA‐AtPRA1 fusion constructs, the PCR products of *AtPRA1* isoforms were cloned into a pUC vector containing the CaMV 35S promoter, an HA tag, a multiple cloning site, and a NOS terminator. The resulting constructs (pUC‐35S::HA‐AtPRA1) were digested with *Xba*I and *Kpn*I restriction endonucleases, and the DNA fragments containing the HA‐AtPRA1 sequence were cloned into the pBIB binary vector digested with *Xba*I and *Kpn*I. FLS2‐GFP, FLS2‐mCherry, and localisation marker constructs were gifts from Robatzek Silk (Addgene #86157 (Robatzek et al. 2006) and #86159 (Mbengue et al. 2016)). All constructs were verified by Sanger sequencing.

### 2.4 Bacterial Inoculation Assay

Flood‐inoculation assays were carried out using 2‐week‐old *Arabidopsis* seedlings grown in 1/2 MS medium, as described previously (Ishiga et al. 2011). Briefly, *Arabidopsis* seedlings in one container were incubated with 30 mL of buffer or bacterial suspension (5 × 10^5^ colony‐forming units [cfu]/mL) supplemented with 0.02% Silwet L‐77 (Lehle Seeds) for 3 min. After removing the buffer or bacterial suspension, plants were transferred to a growth room, and symptoms were observed at 3 days post‐inoculation (dpi) under white or ultraviolet light. Bacterial cell numbers in inoculated plants were determined using serial dilutions (Ishiga et al. 2011). Data were obtained from three biological replicates. Three hypocotyls harvested from separate plates were sterilised for 2 min with 5% hydrogen peroxide and then washed three times with sterile distilled water. The number of cfu was normalised relative to the sample weight (mg). In addition, the bacterial population was determined in more than three independent experiments conducted under the same conditions.

### 2.5 RNA Extraction and Q‐RT‐PCR Analysis

Total RNA was extracted from *Shigella*‐infected leaves (harvested from three plants) using NucleoSpin RNA Plant (MN), according to the manufacturer's protocol. cDNA was synthesised using M‐MLV reverse transcriptase (Invitrogen), according to the manufacturer's instructions. Then, qRT‐PCR was carried out in a CFX Connect Real Time System (BioRad) using iQ SYBR Green Supermix (BioRad) with gene‐specific primers. The transcript levels of target genes were normalised to that of *16 s rRNA* or *Actin*.

### 2.6 Effector Secretion and Virulence Assay

To test effector secretion, pBAV178 was transformed into *Pst* by electroporation (Cadoret et al. 2014). Bacterial suspensions of *Pst* (2.5 × 10^8^ cfu/mL) harbouring the *S. flexneri* 5a effector *VirA* gene were injected into one half of each *Arabidopsis* leaf using a needleless syringe, and cell death was monitored at 1 dpi (Koch et al. 1990). To analyze the virulence of VirA, a bacterial suspension (5 × 10^7^ cfu/mL) of *Pst* containing pBAV179 was used to spray‐inoculate *Arabidopsis* leaves with 0.02% (w/v) Silwet L‐77 or to infiltrate *N. benthamiana* leaves (Moon et al. 2016). After infection, plants were transferred to a growth room. Disease symptoms were monitored at 5 dpi under white light, and bacterial proliferation was assessed at the indicated number of days after infection.

### 2.7 Y2H Screening

Y2H screening was performed by Panbionet Corp. (http://panbionet.com; Pohang, Republic of Korea). Briefly, the full‐length CDS of *VirA* was cloned into pGBKT containing the DNA‐binding domain of GAL4 (bait), and 3.02 × 10^6^ clones from an *Arabidopsis* whole‐seedling cDNA library were cloned into pGADT7 containing the activation domain of GAL4 (prey). Based on two reporter genes (*ADE2* and *HIS3*) under the control of the GAL promoter, different pairs of constructs were co‐transformed into yeast (*Saccharomyces cerevisiae*) strain AHL109, and the yeast transformants harbouring both bait and prey constructs were spread on selection medium (SD‐LW, ‐LWA, or ‐LWH with 5 mM 3‐AT). Constructs from yeast colonies that grew on the selection medium and showed reporter gene expression were analyzed by sequencing and restriction endonuclease digestion. Ten positive colonies were obtained, which contained the following genes: ALB1 (NM_100725), TIM17‐2 (NM_129296), aoc1 (AJ308483), BPA1 (NM_121690), and PRA1.E (NM_100751).

### 2.8 *Agrobacterium‐*Mediated Transient Expression of Genes in *N. benthamiana*

To transiently express the genes of interest in *N. benthamiana*, binary vectors containing the recombinant target gene were transformed into *Agrobacterium tumefaciens* strain GV2260 (Deblaere et al. 1985). The transformed bacteria were grown in LB broth supplemented with appropriate antibiotics at 28°C with shaking at 200 rpm. The cultures were harvested, and bacteria were resuspended in infiltration buffer (10 mM MES [pH 5.6], 10 mM MgCl_2_, and 200 mM acetosyringone [Sigma‐Aldrich]). The optical density of the bacterial suspension measured at 600 nm (OD_600_) was adjusted to 0.4, and the suspension was infiltrated into the leaves of 4‐week‐old *N. benthamiana* plants using a 1‐mL needleless syringe (Moon et al. 2016).

### 2.9 Immunoblotting and Co‐IP Experiments

To perform immunoblotting, total protein extracts were prepared from infected leaves (from three plants) using protein extraction buffer (10 mM HEPES [pH 7.5], 100 mM NaCl, 1 mM EDTA, 10% glycerol, 0.5% Triton X‐100, and 1 × protease inhibitor cocktail [Roche]). Total proteins were separated by 12% SDS‐PAGE and transferred onto a PVDF membrane (Pierce). Antibodies directed against FLAG (Cell Signalling Technology), GFP (Santa Cruz), HA (Sigma‐Aldrich), FLS2 (Agrisera), and mCherry (BioRad) were used for immunoblotting. Target proteins were detected using ECL Plus reagent (GE Healthcare), and signals were captured using an Alliance 9.7 chemiluminescence imaging system (UVITEC).

Co‐IP experiments for GFP‐fusion proteins were performed using GFP‐Trap (Chromotek), according to the manufacturer's protocol. Immunoprecipitated proteins were analyzed by immunoblotting with appropriate antibodies.

### 2.10 Bimolecular Fluorescence Complementation (BiFC) Experiments and Subcellular Localisation Analysis

To perform BiFC experiments, the CDSs of VirA, PRA1.E, PRA1. F3, and PRA1.F4 were cloned into pSPYNE‐35S and pSPYCE‐35S vectors (Walter et al. 2004). For subcellular localisation and co‐localisation analyses, the CDSs of VirA and PRA1 isoforms were fused to the N‐terminus of GFP or mRFP in appropriate expression vectors. Previously published organelle marker constructs fused to GFP or YFP were used as references (Lee et al. 2017). All GFP‐, mRFP‐, and YFP‐tagged constructs were introduced into *A. tumefaciens* strain GV2260. Agrobacterial suspensions (OD_600_ = 0.4) were infiltrated into the leaves of 4‐week‐old *N. benthamiana* plants. Fluorescence signals were detected using a Nikon C2 confocal laser scanning microscope (Nikon) with 30× or 60× objectives at the following excitation and emission wavelengths: YFP (λ_ex_ = 514 nm, λ_em_ = 520–550 nm), GFP (λ_ex_ = 488 nm, λ_em_ = 505–530 nm), mCherry/mRFP (λ_ex_ = 561 nm, λ_em_ = 570–620 nm). For each experiment, three leaves from three independent plants were infiltrated, and at least three microscopic fields per leaf were examined, including control samples.

### 2.11 Generation of Transgenic Plants

To generate transgenic plants constitutively overexpressing *AtPRA1* isoforms, the HA*‐AtPRA1* fusions were cloned into the pBIB binary vector. ProPRA1.F3::HA*‐PRA1.F3* was constructed by separately amplified PCR fragments including the PRA1.F3 promoter, a HA tag, and the *PRA1.F3* open reading frame with a stop codon, and then cloned into the *Hind*Ⅲ and *Sal*Ⅰ sites of the pCAMBIA 1390 binary vector. The resulting vectors were transformed into *Agrobacterium* and then introduced into *Arabidopsis* plants using the floral‐dip method (Clough and Bent 1998). Transformants were screened on 1/2 MS plates supplemented with hygromycin B (50 mg/L). At least 10 independent events were selected from each transformation in the T1 generation, and homozygous plants were identified in the T2 generation. Homozygous T3 plants were used for immunoblot analysis using an anti‐HA antibody.

### 2.12 Membrane Protein Fractionation

Total membrane proteins, PM proteins, and intracellular organelle membrane proteins were extracted from agroinfiltrated *N. benthamiana* plants at 48 h post‐inoculation (hpi) using a Minute Plasma Membrane Protein Isolation Kit for Plants (Invent Biotechnologies), as previously described (Shen et al. 2017). H^+^‐ATPase was detected with an anti‐H^+^‐ATPase antibody (Agrisera). The cytosolic fraction was detected by staining the PVDF membrane with Ponceau S (PS).

### 2.13 MAPK Activation Analysis

flg22 peptide (Alpha Diagnostic Intl. Inc.) was used to measure plant MAPK activity (Bethke et al. 2009). Briefly, 10‐day‐old *Arabidopsis* seedlings were transferred to a 12‐well plate, with each well containing 2 mL of water for recovery overnight. Subsequently, the seedlings were treated with 100 nM flg22 for 0, 15, and 45 min (Li et al. 2019). Samples were collected and ground in phosphoprotein extraction buffer (50 mM Tris‐HCl [pH 7.5], 150 mM NaCl, 5 mM EDTA, 0.1% Triton X‐100, 1 × protease inhibitor cocktail, 1 mM DTT, 2 mM NaF, and 2 mM Na_2_VO_3_) for protein extraction. Phosphorylation of MPK3 and MPK6 was examined by immunoblotting with anti‐phospho‐p44 and p42 MAPK (ERK1/2) antibodies (Cell Signalling Technology), respectively.

### 2.14 Chemical Inhibitor Treatment

To inhibit 26S proteasomal or autophagic degradation of PRA1 isoforms, the agroinfiltrated sites of *N. benthamiana* leaves were treated with 50 μM MG132 (Sigma‐Aldrich) or 1 μM ConA (Sigma‐Aldrich), respectively, at 1 dpi. Leaves were sampled at 12 h after chemical treatment, and immunoblot analysis was performed using total proteins extracted from the treated leaf areas.

### 2.15 Ubiquitination Analysis

To examine ubiquitination of PRA1 isoforms in planta, *N. benthamiana* leaves were co‐infiltrated with VirA*‐*GFP and HA‐PRA1 isoforms. The agroinfiltrated leaves were treated with 50 μM MG132 at 12 h before sampling (Shen et al. 2016). Total protein extracts were prepared using IP lysis buffer (10 mM Tris‐HCl [pH 7.5], 150 mM NaCl, 0.5 mM EDTA, 0.5% NP‐40, and 1 × protease inhibitor cocktail), and HA‐PRA1 isoforms were immunoprecipitated using Pierce Anti‐HA Magnetic Beads (Thermo scientific). Polyubiquitination was detected with an anti‐ubiquitin antibody (Santa Cruz).

### 2.16 Statistical Analysis

Statistical analyses were performed using GraphPad Prism software (Version 8), and data were represented as mean ± standard deviation (SD). The statistical significance of data was examined using a one‐way analysis of variance (ANOVA; SPSS v.18, IBM) or the one‐sided unpaired Student's t‐test (Microsoft Office Excel). Significant differences (*p* < 0.05) among samples were indicated using letters or asterisks. Detailed statistical information is provided in the figure legends. “*n*” represents the number of samples in one biological repeat. Experiments were repeated at least three times unless otherwise stated.

### 2.17 Accession Numbers

VirA (UniProt: Q7BU69), PRA1.E (AT1G08770; NM_100751), PRA1.F3 (AT3G13720; NM_112222), PRA1.F4 (AT3G13710; NM_112221), PRA1.B5 (AT5G01640; NM_120242), PRA1.G1 (AT1G55640; NM_104440), FLS2 (AT5G46330; NM_ 001344672), 16S rRNA (GenBank: X96963), Actin2 (AT3G18780; NM_001338359), WRKY29 (AT4G23550; NM_ 118486), FRK1 (AT2G19190; NM_127476), and PR1 (AT2G14610; NM_127025).

## 3 Results

### 3.1 Virulence Activity of the *Shigella* VirA Effector Is Conserved in Plants

**Figure 1:**
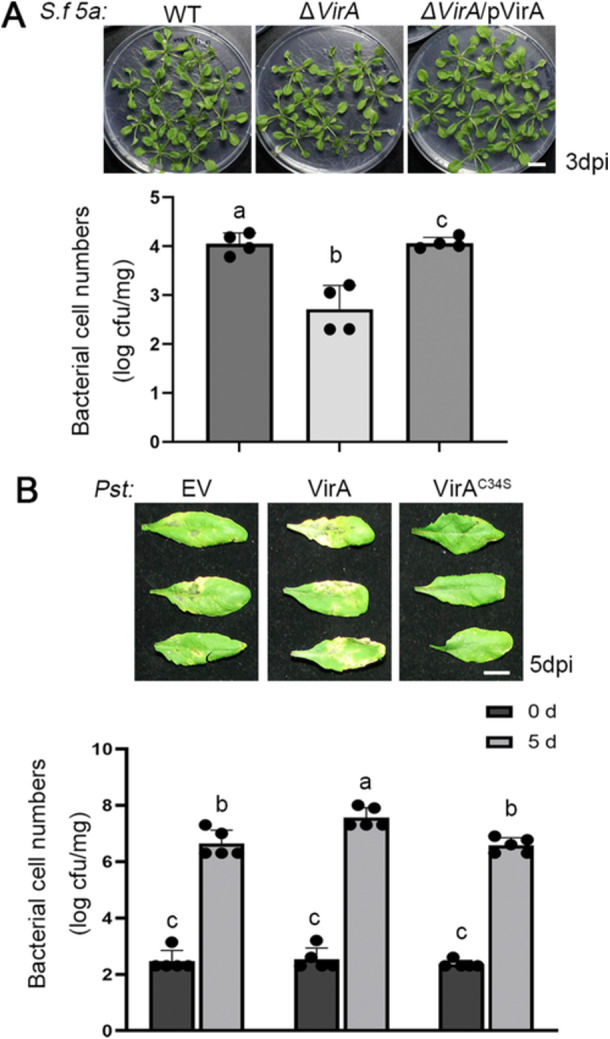
Virulence activity of *Shigella* VirA in *Arabidopsis*. (A) VirA promotes *Shigella* virulence in *Arabidopsis*. Two‐week‐old seedlings were flood‐inoculated with *S. flexneri* 5a WT, ΔvirA, or ΔvirA complemented with pVirA at 5 × 10⁵ cfu/mL. Representative disease symptoms were photographed (top), and bacterial proliferation was quantified at 3 dpi (*n* = 4) (bottom). (B) Expression of WT VirA, but not VirA^C34S^, enhances *Pst* virulence. Leaves were spray‐inoculated with *Pst* carrying an EV, VirA, or VirA^C34S^ at 5 × 10⁷ cfu/mL. Representative disease symptoms were photographed at 5 dpi (top), and bacterial populations were quantified at 0 and 5 dpi (*n* = 5) (bottom). Data are means ± SD. Statistical significance was determined by one‐way or two‐way ANOVA followed by Tukey's HSD test (*p* < 0.05). Different letters indicate statistically significant differences.

VirA is a T3SS effector essential for *Shigella* virulence in mammalian hosts (Uchiya et al. 1995). To assess whether VirA also contributes to virulence in plants, we generated a VirA‐deletion mutant (ΔvirA) in *Shigella flexneri* serotype 5a strain M90T (*S. flexneri* 5a), which is a widely used model for T3SS studies (Onodera et al. 2012; Pakbin et al. 2021). The ΔvirA mutant displayed normal in vitro growth and T3SS effector secretion, similar to the wild‐type (WT) and in contrast to the T3SS‐deficient control strain (BS17642) (Figure S1A,B). However, its proliferation in *Arabidopsis thaliana* was significantly impaired (~10‐fold), supporting a role for VirA in promoting bacterial fitness in planta (Figure 1A). Expression of *VirA* in planta during *Shigella* inoculation was validated by qRT‐PCR (Figure S1C).

To further examine the virulence potential of VirA in a plant‐specific context, we tested its activity in a phytopathogenic background. To determine whether VirA can be delivered into plant cells by a heterologous T3SS, we fused it to the C‐terminal AvrRpt2^101–255^ domain, which is a well‐established T3SS reporter that induces a hypersensitive response (HR) upon translocation (Vinatzer et al. 2005). Infiltration of *Arabidopsis* leaves with *Pseudomonas syringae* pv. tomato DC3000 (*Pst*) expressing the VirA‐AvrRpt2^101–255^ fusion triggered a HR comparable to that induced by *Pst* AvrRpt2, indicating that VirA is efficiently translocated into plant cells via the *Pst* T3SS (Figure S1D).

We next investigated whether VirA enhances the virulence of a plant‐adapted pathogen. Expression of VirA in *Pst* resulted in more severe disease symptoms and significantly increased bacterial growth in *Arabidopsis* compared with the empty vector (EV) control (Figure 1B). By contrast, expression of the VirA^C34S^ mutant, in which the conserved cysteine is substituted by serine and which lacks virulence activity in mammalian cells (Yoshida et al. 2006; Germane et al. 2008), did not enhance virulence, showing symptom development and bacterial titre comparable to those of the EV control (Figure 1B). Similar results were observed in *N. benthamiana* (Figure S2A,B), indicating that VirA retains virulence activity in plant hosts and enhances pathogenicity in a manner dependent on its conserved functional residue.

### 3.2 VirA Interacts With *Arabidopsis* PRA1 in a C34‐Dependent and Membrane‐Associated Manner

To identify host targets of VirA, we performed a Y2H screen using an *Arabidopsis* cDNA library with VirA as bait. Under stringent selection conditions, a total of ten His⁺/Ade⁺ positive clones were recovered. Sequence analysis revealed that all ten clones encoded inframe proteins, which were classified into five distinct *Arabidopsis* genes based on functional annotation and predicted molecular features (Table S3). To evaluate their relevance in planta, each candidate protein was transiently expressed in plant cells and tested for interaction with VirA by Co‐IP. Among the five candidates, only PRA1.E reproducibly co‐immunoprecipitated with VirA, identifying PRA1.E as a bona fide VirA‐interacting protein in plant cells and leading us to select it for further functional characterisation.

**Figure 2:**
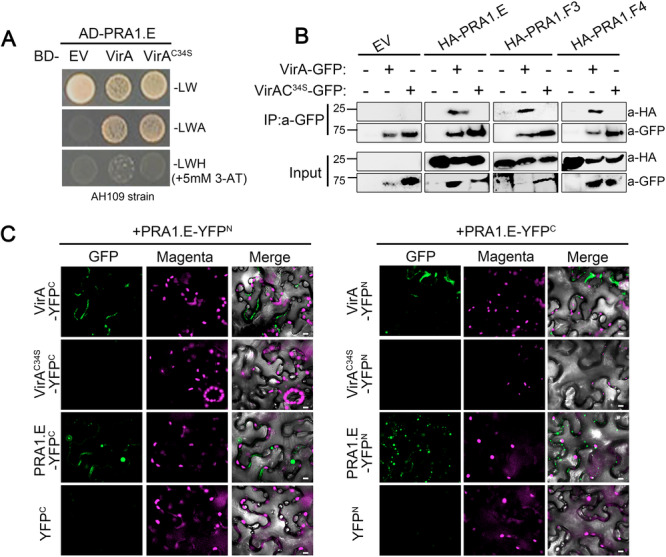
VirA interacts with PRA1 isoforms in a membrane localisation‐dependent manner. (A) Y2H assay showing the interaction between VirA and PRA1.E. The AD‐fused PRA1.E was co‐expressed with BD‐fused EV, VirA or VirA^C34S^ in *Saccharomyces cerevisiae* strain AH109. Transformants were plated on selective medium lacking leucine and tryptophan (SD‐LW), leucine, tryptophan, and adenine (SD‐LWA), or leucine, tryptophan, and histidine (SD‐LWH) supplemented with 5 mM 3‐AT. (B) Co‐IP assay confirming in planta interactions between VirA and PRA1 isoforms. Proteins were transiently expressed in *N. benthamiana* and immunoprecipitated using GFP‐Trap. (C) BiFC assay validating the interaction between VirA and PRA1.E. Reconstituted YFP fluorescence was visualised in epidermal cells at 48 hpi. Magenta signals represent chlorophyll autofluorescence. Scale bars = 10 µM. [Color figure can be viewed at wileyonlinelibrary.com]

As shown in Figure 2A, Y2H assays demonstrated that PRA1.E interacted with both WT VirA and the VirA^C34S^ mutant in yeast. In contrast, Co‐IP assays performed in *N. benthamiana* revealed that PRA1.E co‐immunoprecipitated with WT VirA but not with the VirA^C34S^ mutant, indicating that the conserved C34 residue is required for the interaction in planta (Figure 2B). Notably, the VirA^C34S^ mutant retained its ability to interact with PRA1.E in yeast (Figure 2A), highlighting a system‐dependent difference in interaction requirements.

We next validated the VirA‐PRA1.E interaction in planta using BiFC. Co‐expression of PRA1.E and WT VirA reconstituted YFP fluorescence in epidermal cells, whereas co‐expression of PRA1.E and VirA^C34S^ did not (Figure 2C). The YFP signal was not dependent on the orientation of the YFP fusion. As a positive control, PRA1.E‐YFP^N^ and PRA1.E‐YFP^C^ co‐expression produced strong YFP signals, consistent with PRA1 oligomerization (Alvim Kamei et al. 2008).

We hypothesised that the differential interactions of VirA^C34S^ observed between yeast and plant systems may be attributable to differences in subcellular localisation. Notably, expression of VirA^C34S^‐GFP in *N. benthamiana* consistently induced HR‐like cell death, which precluded reliable analysis of its steady‐state subcellular localisation by live‐cell imaging (Figure S2). Therefore, subcellular fractionation assays were performed at 1 dpi, prior to the appearance of visible symptoms. Under these conditions, VirA‐GFP was strongly enriched in the membrane fraction, whereas VirA^C34S^‐GFP was detected almost exclusively in the cytosolic fraction (Figure S3A).

Consistent with these findings, confocal microscopy analysis of cells co‐expressing VirA^C34S^‐GFP and the early endosomal marker mCherry‐Rab5 showed little to no colocalization, with VirA^C34S^‐GFP displaying a diffuse cytosolic fluorescence pattern; however, imaging was constrained by cell collapse (Figure S3B). In contrast, VirA‐GFP localised to multiple endomembrane compartments, including the endoplasmic reticulum, the trans‐Golgi network, and early endosomes, as confirmed by colocalization with compartment‐specific markers (Figure S3C) (Ivanov and Harrison 2014). Collectively, these results demonstrate that the conserved C34 residue is required for stable membrane association of VirA in planta, supporting the conclusion that VirA‐PRA1 interactions occur within membrane compartments.

### 3.3 VirA Specifically Targets *Arabidopsis* PRA1.F3 to Modulate Immunity

Although PRA1.E emerged as a VirA interactor in the initial screen, the *Arabidopsis* PRA1 family comprises multiple isoforms with distinct expression patterns and subcellular localisations. To assess the isoform selectivity of VirA in planta, we analyzed five representative isoforms, PRA1.B5, PRA1.G1, PRA1.E, PRA1.F3, and PRA1.F4, selected based on their diverse expression profiles and intracellular distributions (Alvim Kamei et al. 2008) (Figure S4A). Corresponding T‐DNA insertion mutants were obtained from the Arabidopsis Stock Center, and transgenic lines overexpressing the respective *PRA1* genes were generated as described previously (Lee et al. 2011; Lee et al. 2017) (Table S1).

**Figure 3:**
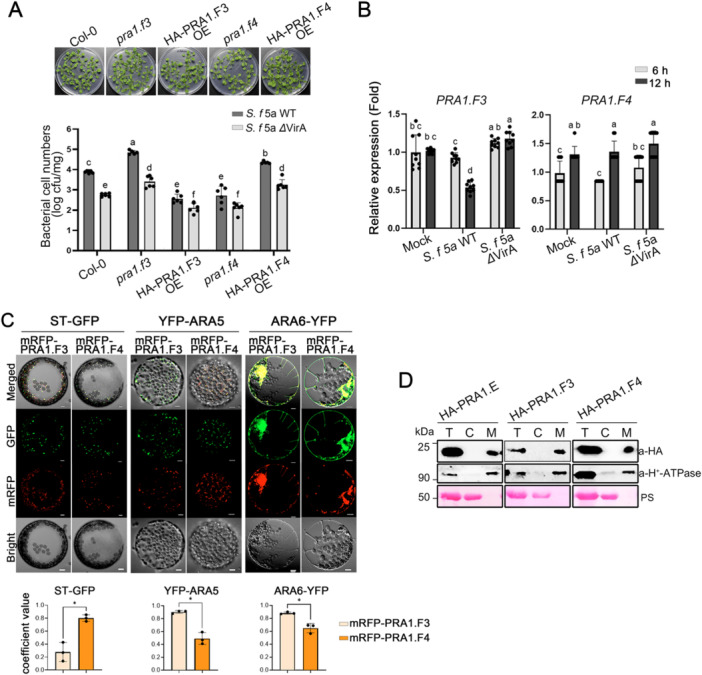
PRA1.F3 is the functional target of VirA among *Arabidopsis* PRA1 isoforms. (A) PRA1.F3 promotes resistance while PRA1.F4 enhances susceptibility to *S. flexneri 5a*. *Arabidopsis* lines were flood‐inoculated with *S. flexneri 5a* WT or Δ*virA* strains at 5 × 10⁵ cfu/mL. Representative disease symptoms were photographed (top), and bacterial proliferation was quantified at 3 dpi (bottom). Data represent means ± SD (*n* = 6). Different letters indicate significant differences (one‐way ANOVA with Tukey's HSD, *p* < 0.05). (B) Transcript levels of PRA1 isoforms after *S. flexneri* 5a infection. *Arabidopsis* seedlings were flood‐inoculated with *S. flexneri* 5a WT or ΔvirA at a concentration of 5 × 10^5^ cfu/ml. *PRA1.F3* and *PRA1.F4* transcript levels were quantified at 6 and 12 hpi by qRT‐PCR and normalised to *Actin*. Data represent means ± SD (*n* = 9). Statistically significant differences were determined by two‐way ANOVA followed by Tukey's HSD (*p* < 0.05). (C) Subcellular localisation of PRA1.F3 and PRA1.F4 in *N. benthamiana*. mRFP‐tagged PRA1 isoforms were co‐expressed with organelle markers: ST‐GFP (Golgi), YFP‐ARA5 (TGN/early endosome), and ARA6‐YFP (late endosome). Confocal images were captured at 2 dpi. Scale bars = 10 µM. Pearson's correlation coefficients (right) quantify co‐localisation between red and green channels (mean ± SD, *n* = 3). Asterisks indicate significant differences as determined by Student's *t*‐test (*p* < 0.05). (D) Membrane association of PRA1 isoforms assessed by subcellular fractionation. *N. benthamiana* leaves were infiltrated with *Agrobacterium* carrying HA‐PRA1.E, HA‐PRA1.F3, or HA‐PRA1.F4 constructs. At 2 dpi, total protein extracts were fractionated into membrane (M) and soluble/cytosolic (C) fractions and analyzed by immunoblotting using an anti‐HA antibody. PM enrichment was verified using an anti‐H⁺‐ATPase antibody, and PS staining of RuBisCO served as a loading control. T, total protein fraction. [Color figure can be viewed at wileyonlinelibrary.com]

Infection assays with *S. flexneri* 5a revealed that bacterial proliferation was significantly increased in *pra1.e* and *pra1.f3* mutants, whereas it was reduced in the *pra1.f4* mutant compared with Columbia‐0 (Col‐0) WT plants (Figure 3A and S4B). Conversely, transgenic plants overexpressing HA‐tagged PRA1.F3 (HA‐PRA1.F3 OE) restricted bacterial growth, whereas HA‐PRA1.F4 OE plants exhibited enhanced susceptibility. In contrast, HA‐PRA1.E OE plants showed susceptibility levels comparable to Col‐0, indicating that PRA1.E overexpression does not substantially alter basal immunity (Figure S4B). Together, these results indicate that PRA1.F3 acts as a positive regulator of basal immunity, whereas PRA1.F4 functions as a negative regulator.

To determine whether these isoforms contribute to VirA‐mediated immune suppression, we performed infection assays using a *virA* deletion mutants (ΔvirA) (Figure 3A). The increased susceptibility observed in *pra1.f3* and HA‐PRA1.F4 OE plants was significantly attenuated upon ΔvirA infection, indicating that these immune phenotypes are at least partly dependent on VirA activity. Notably, both *pra1.f3* and HA‐PRA1.F4 OE plants remained more susceptible than Col‐0 even in the absence of VirA, suggesting that PRA1.F3 and PRA1.F4 also participate in basal immune regulation independently of VirA.

Given the contrasting immune phenotypes associated with PRA1.F3 and PRA1.F4, we next examined whether VirA selectively regulates their expression. Quantitative RT‐PCR analysis showed that *PRA1.F3* transcript levels were significantly reduced following infection with WT *Shigella*, whereas no such reduction was observed in plants infected with ΔvirA, indicating that VirA specifically represses *PRA1.F3* expression (Figure 3B). In contrast, *PRA1.F4* and other members of the PRA1.F clade (*PRA1.F1*, *PRA1.F2*, and *PRA1.E*) showed no significant transcriptional changes upon *Shigella* infection (Figures 3B and S4C), further supporting the selective targeting of PRA1.F3 by VirA.

To exclude potential developmental effects, we examined the mutant and overexpression lines under standard growth conditions. No obvious morphological differences were observed compared with Col‐0 (Figure S5A). Quantitative RT‐PCR confirmed appropriate expression of the target genes in all lines without compensatory changes between *PRA1.F3* and *PRA1.F4* (Figure S5B). The *pra1.f4* mutant used in this study corresponds to a previously characterised T‐DNA insertion line in which the insertion disrupts the coding region of PRA1.F4, preventing the production of a functional protein (Lee et al. 2017). As reported previously, *PRA1.F4* transcript levels in this mutant are comparable to those in Col‐0, indicating that the T‐DNA insertion affects protein function rather than transcript accumulation.

To investigate the mechanistic basis underlying the contrasting roles of PRA1.F3 and PRA1.F4 during *Shigella* inoculation, we compared their subcellular localisation and interaction with VirA. Co‐IP assays in *N. benthamiana* confirmed that both PRA1.F3 and PRA1.F4 interacted with WT VirA but not with VirA^C34S^ mutant, reinforcing the importance of the conserved C34 residue for binding in planta (Figure 2B). Consistent with these results, BiFC assays further demonstrated that both PRA1.F3 and PRA1.F4 interact with WT VirA in plant cells, whereas no YFP signal was detected when either isoform was co‐expressed with VirA^C34S^ (Figure S6).

Subcellular localisation analysis revealed that PRA1.F3 was co‐localised with the endosomal markers ARA5 and ARA6 (Ebine et al. 2012; Heard et al. 2015), indicating its presence in endosomes, whereas PRA1.F4 was co‐localised with ST‐GFP, a marker for the Golgi apparatus (Lee et al. 2017) (Figure 3C). These localisation patterns were further confirmed in intact *N. benthamiana* epidermal cells, excluding potential artifacts associated with protoplast‐based assays (Figure S7). Membrane fractionation analysis showed that both PRA1.F3 and PRA1.F4 were associated with membrane compartments (Figure 3D).

Collectively, these results demonstrate that although VirA is capable of interacting with multiple PRA1 isoforms in planta, PRA1.F3 represents the primary functional target of VirA due to its endosomal localisation and role in immune receptor trafficking. Through selective repression of PRA1.F3 expression and interference with its function, VirA effectively modulates host immunity and promotes bacterial virulence.

### 3.4 PRA1.F3 and PRA1.F4 Oppositely Regulate Plant Immunity and FLS2 Receptor Accumulation at the PM

**Figure 4:**
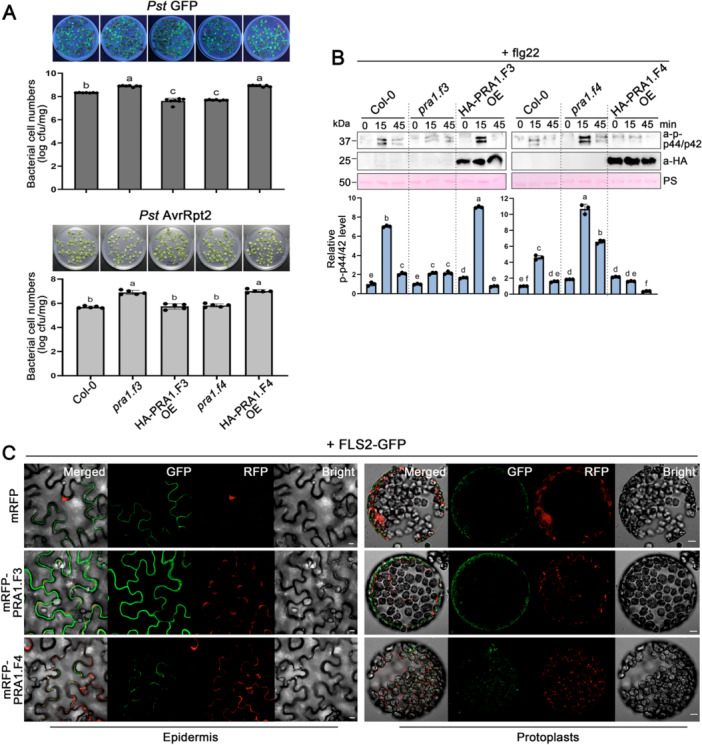
PRA1.F3 and PRA1.F4 oppositely regulate FLS2 trafficking and plant immunity. (A) PRA1 isoforms differentially modulate immune responses against compatible and incompatible bacterial pathogens. *Arabidopsis* seedlings of the indicated genotypes were flood‐inoculated with *Pst* expressing GFP (Pst GFP; compatible) or AvrRpt2 (*Pst* AvrRpt2; incompatible). Representative disease symptoms are shown, and bacterial proliferation was quantified at 3 dpi. Data represent means ± SD (*n* = 7 for *Pst* GFP; *n* = 5 for *Pst* AvrRpt2). (B) PRA1.F3 enhances, whereas PRA1.F4 suppresses, flg22‐triggered MAPK activation. Ten‐day‐old seedlings were treated with 100 nM flg22, and protein samples were collected at the indicated time points. Phosphorylated MAPKs were detected by immunoblotting using anti‐phospho‐p44/42 MAPK antibody. Relative phospho‐p44/42 MAPK band intensities were quantified using ImageJ. Statistical significance was determined by one‐way ANOVA with Tukey's HSD test (*p* < 0.05). (C) PRA1 isoforms differentially affect FLS2 subcellular localisation. FLS2‐GFP was co‐expressed with mRFP, mRFP‐PRA1.F3, or mRFP‐PRA1.F4 in *N. benthamiana* leaves. Fluorescence signals were observed in intact epidermal cells (left) and isolated leaf protoplasts (right) at 2dpi by confocal microscopy. Scale bars = 10 µM. [Color figure can be viewed at wileyonlinelibrary.com]

To assess whether the opposing immune roles of PRA1.F3 and PRA1.F4 extend beyond *Shigella* infection, we challenged mutant and OE lines with *Pseudomonas syringae* strains representing both basal and ETI‐inducing pathogens. GFP‐labelled *Pst* was used as a compatible pathogen (Wang et al. 2007), whereas *Pst* carrying AvrRpt2, an avirulence factor recognised by the NB‐LRR protein RPS2, served as an incompatible pathogen (Whalen et al. 1991). Upon infection with *Pst* GFP, *pra1.f3* and *pra1.f4* mutants displayed increased susceptibility and resistance, respectively, while the OE lines exhibited the opposite phenotypes (Figure 4A). Infection with *Pst* AvrRpt2 further revealed that the RPS2‐mediated ETI response was attenuated in *pra1.f3* mutants and in HA‐PRA1.F4 OE plants (Figure 4A). These results indicate that PRA1.F3 and PRA1.F4 play antagonistic roles in both basal and ETI.

We next examined their involvement in PTI signalling by monitoring MAPK activation after flg22 treatment (Bethke et al. 2009). Immunoblotting with anti‐phospho‐p44/42 MAPK antibodies revealed that MAPK phosphorylation was reduced in *pra1.f3* and HA‐PRA1.F4 OE plants relative to Col‐0, but was enhanced in HA‐PRA1.F3 OE and *pra1.f4* plants (Figure 4B). Thus, PRA1.F3 promotes, whereas PRA1.F4 suppresses, early PTI signalling through MAPK activation.

Given their contrasting immune roles and known functions in membrane trafficking (Liang and Li 2000; Liu et al. 2006; Alvim Kamei et al. 2008), we tested whether PRA1 isoforms modulate trafficking of FLS2, a key immune receptor for flg22 perception (Yuan et al. 2021). FLS2‐GFP was co‐expressed with mRFP‐tagged PRA1.F3 or PRA1.F4 in *N. benthamiana* leaves (Robatzek et al. 2006). Confocal microscopy revealed that PRA1.F3 enhanced, whereas PRA1.F4 reduced, FLS2 accumulation at the PM (Figure 4C). Biochemical fractionation corroborated these observations, showing that FLS2‐GFP was enriched in the PM fraction in the presence of PRA1.F3 but redistributed to intracellular membrane fractions when co‐expressed with PRA1.F4 (Figure S8A,B). To determine whether these effects were attributable to transcriptional regulation, we quantified *FLS2* transcript levels in *pra1* mutants and OE lines. qRT‐PCR analysis showed no significant differences among genotypes (Figure S8C), indicating that PRA1.F3 and PRA1.F4 regulate FLS2 at the post‐transcriptional level.

To assess whether PRA1.F3 broadly regulates PM accumulation of PRRs, we examined the elongation factor Tu receptor EFR as an additional PRR. In contrast to FLS2, EFR‐GFP accumulation was not significantly altered by co‐expression of PRA1.F3 or PRA1.F4 (Figure S9A). Consistent with this observation, biochemical fractionation analyses showed that co‐expression of PRA1.F3 or PRA1.F4 with EFR‐GFP in *N. benthamiana* resulted in only a modest increase in EFR‐GFP levels in the PM (Figures S9B,C), without a corresponding change in overall subcellular distribution. These findings indicate that PRA1.F3‐mediated regulation is not a general feature of PRR accumulation.

Given that PRA1.F3 selectively regulates FLS2 but not EFR accumulation at the PM, we next examined whether loss of FLS2 or EFR differentially affects susceptibility to *Shigella* infection (Figure S9D). As a control, we first assessed susceptibility to the *Pst*. Consistent with the established roles of both receptors in antibacterial immunity, *fls2* and *efr* mutant plants exhibited enhanced susceptibility to *Pst* compared with Col‐0. In contrast, infection with *S. f* 5a revealed a distinct pattern: bacterial proliferation was significantly increased in *fls2* mutants, whereas *efr* mutants displayed susceptibility levels comparable to those of Col‐0. These results indicate that, unlike *Pst*, *Shigella* virulence in plants predominantly depends on FLS2 but not EFR.

### 3.5 The GTPase‐Activating Activity of VirA Is Required for Suppression of FLS2 Accumulation and Plant Immunity

VirA harbours a Tre‐2/Bub2/Cdc16 (TBC)‐like domain and functions as a GAP for Rab1 in animal systems (Dong et al. 2012). To determine whether GAP activity is also required for VirA function in plants, we introduced point mutations in two conserved catalytic residues, R188 and Q280, generating VirA^R188K^, VirA^Q280A^, and the double mutant VirA^RQ^ (Figure S10A). These constructs were expressed in *Pst*, and protein accumulation was confirmed by immunoblotting (Figure S10B). All GAP‐deficient variants exhibited significantly reduced virulence in *Arabidopsis* seedlings compared with WT VirA, with no further reduction in the double mutant (Figure S10C), indicating that GAP activity is essential for VirA‐mediated virulence.

We next examined whether disruption of GAP activity affected VirA subcellular localisation or interaction with PRA1 isoforms. Subcellular fractionation revealed that the GAP‐deficient mutants remained associated with membrane fractions and displayed increased protein stability relative to WT VirA (Figure S10D). Co‐IP assays demonstrated that they retained binding to both PRA1.F3 and PRA1.F4 (Figure S10E), indicating that GAP activity is dispensable for membrane association and PRA1 binding.

**Figure 5:**
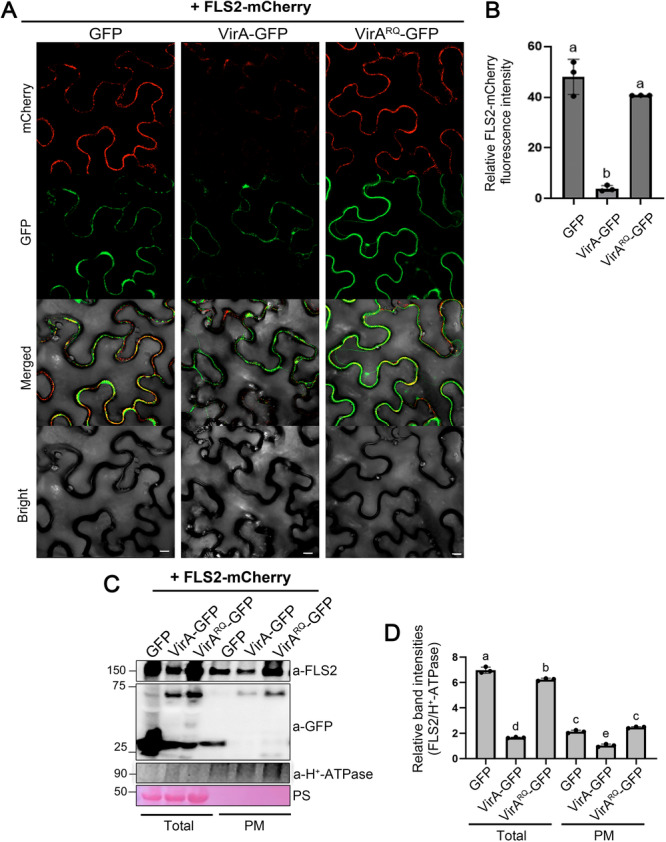
GAP activity of VirA is required to suppress FLS2 trafficking. (A) VirA suppresses FLS2 localisation to the PM in a GAP activity‐dependent manner. FLS2‐mCherry was co‐expressed with GFP (control), VirA‐GFP, or VirA^RQ^‐GFP in *N. benthamiana*. Confocal images were acquired at 2 dpi. Scale bars = 10 µM. (B) Quantification of FLS2‐mCherry fluorescence at the PM. Mean TRITC channel intensities were measured from confocal images using NIS‐Elements AR software. Quantification was performed using representative images from independent experiments, and mean values were subjected to statistical analysis (one‐way ANOVA followed by Tukey's HSD test, *p* < 0.05). (C) Subcellular fractionation of FLS2‐mCherry. Total protein extracts from samples described in (A) were fractionated to isolate plasma PM fractions. FLS2 distribution was analyzed by immunoblotting using an anti‐mCherry antibody. (D) Densitometric analysis of PM‐localised FLS2. Relative FLS2‐mCherry band intensities were quantified using ImageJ and normalised to the PM marker H^+^‐ATPase. Statistical significance was determined by one‐way ANOVA followed by Tukey's HSD test (*p* < 0.05). [Color figure can be viewed at wileyonlinelibrary.com]

To directly assess the functional importance of GAP activity in regulating FLS2, we co‐expressed FLS2‐mCherry with either WT VirA or the GAP‐deficient VirA^RQ^ mutant in *N. benthamiana*. While WT VirA markedly reduced FLS2‐mCherry fluorescence at the PM, VirA^RQ^ had no effect (Figure 5A). Quantification of FLS2‐mCherry fluorescence intensity at the PM confirmed a significant reduction in the presence of WT VirA but not VirA^RQ^ (Figure 5B). Biochemical fractionation analyses further corroborated these results, showing that both PM‐localised and total FLS2 protein levels were decreased upon expression of WT VirA, but remained unchanged when co‐expressed with VirA^RQ^ (Figure 5C,D). Collectively, these results support a sequential mechanism in which VirA first associates with PRA1 isoforms at endomembrane compartments and subsequently employs its GAP activity to suppress FLS2 accumulation at the plasma membrane, thereby attenuating downstream immune signalling.

### 3.6 VirA Targets PRA1.F3 for Proteasomal Degradation to Suppress FLS2 Accumulation and Immunity

**Figure 6:**
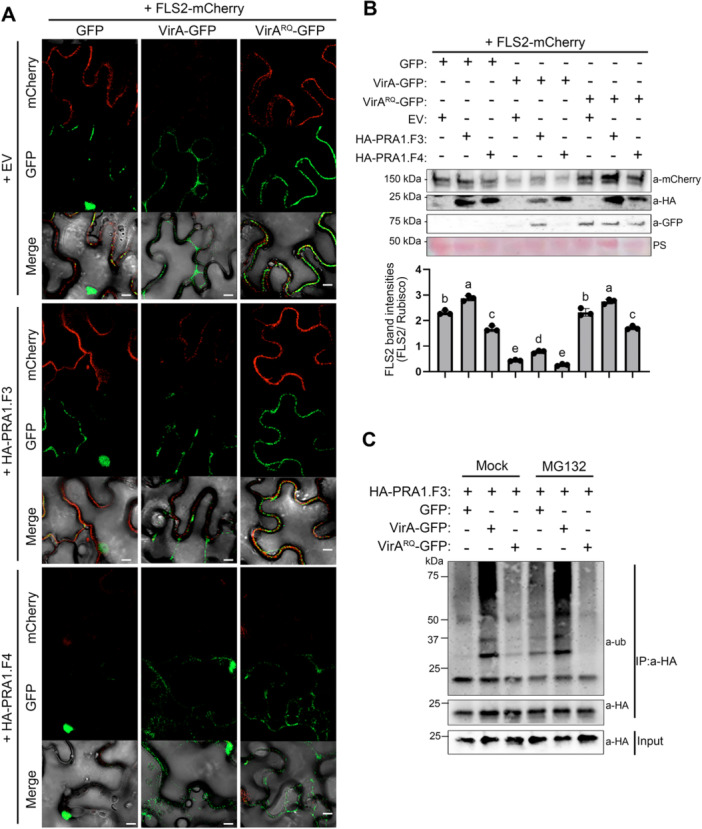
VirA suppresses PRA1.F3‐mediated FLS2 trafficking via proteasome‐dependent degradation. (A) VirA interferes with PRA1.F3‐mediated enhancement of the PM localisation of FLS2. FLS2‐mCherry was co‐expressed with HA‐tagged PRA1 isoforms and WT VirA‐GFP or the GAP‐inactive mutant VirA^RQ^‐GFP in *N. benthamiana* leaves. The subcellular localisation of FLS2‐mCherry was visualised by confocal microscopy at 2 dpi. Scale bars = 10 µM. (B) VirA reduces PRA1.F3 and FLS2 protein abundance in a GAP activity‐dependent manner. Total protein extracts corresponding to the samples shown in (A) were subjected to immunoblot analysis using anti‐mCherry (FLS2) and anti‐HA (PRA1 isoforms) antibodies. Relative FLS2 band intensities were quantified using ImageJ and normalised to the loading control, and statistical significance was determined by one‐way ANOVA followed by Tukey's HSD test (*p* < 0.05). (C) VirA promotes ubiquitination of PRA1.F3 in planta. *N. benthamiana* leaves co‐expressing HA‐PRA1.F3 with WT VirA‐GFP or VirA^RQ^‐GFP were treated with 50 µM MG132 at24 hpi. Total protein extracts were prepared 12 h after treatment and subjected to immunoprecipitation using anti‐HA magnetic beads. Ubiquitinated PRA1.F3 was detected by immunoblotting with an anti‐ubiquitin antibody. [Color figure can be viewed at wileyonlinelibrary.com]

To explore how VirA interferes with PRA1.F3‐dependent regulation of FLS2 accumulation, we co‐expressed FLS2‐mCherry with PRA1.F3 or PRA1.F4 in *N. benthamiana* in the presence or absence of WT VirA or the GAP‐deficient mutant VirA^RQ^ (Figure 6A). Consistent with previous observations, PRA1.F3 enhanced, whereas PRA1.F4 reduced, FLS2‐mCherry accumulation at the PM when co‐expressed with GFP alone. Co‐expression of VirA‐GFP abolished the PRA1.F3‐mediated enhancement of FLS2‐mCherry accumulation, whereas VirA^RQ^‐GFP had no effect. By contrast, PRA1.F4‐mediated suppression of FLS2 accumulation was unaffected by either VirA variant.

Immunoblot analysis further revealed that WT VirA specifically reduced the protein levels of PRA1.F3 and FLS2, but not PRA1.F4, and that this effect was dependent on GAP activity (Figure 6B). FLS2 band intensities were quantified by densitometric analysis and normalised to the Rubisco loading control, with the relative values shown below the blots. To identify the degradation pathway involved, infiltrated tissues were treated with the proteasome inhibitor MG132 or the autophagy inhibitor concanamycin A (ConA) (Huss et al. 2002; Bassham 2015). MG132, but not ConA, effectively blocked VirA‐induced degradation of PRA1.F3 and FLS2, indicating that this process is mediated by the ubiquitin‐proteasome system (UPS) (Figure S11A). In support of this conclusion, PRA1.F3 exhibited increased ubiquitination when coexpressed with WT VirA, but not with VirA^RQ^, and this modification was further enhanced upon MG132 treatment (Figure 6C). By contrast, PRA1.F4 displayed only a slight increase in ubiquitination with WT VirA, and this was evident only under MG132 treatment (Figure S11B), suggesting a much weaker and less consistent modification. Collectively, these findings demonstrate that VirA specifically targets PRA1.F3 for UPS‐mediated degradation via its GAP activity, thereby destabilising FLS2 accumulation at the PM and attenuating host immune signalling.

### 3.7 PRA1.F3 Repression by VirA Impairs Plant Immunity

**Figure 7:**
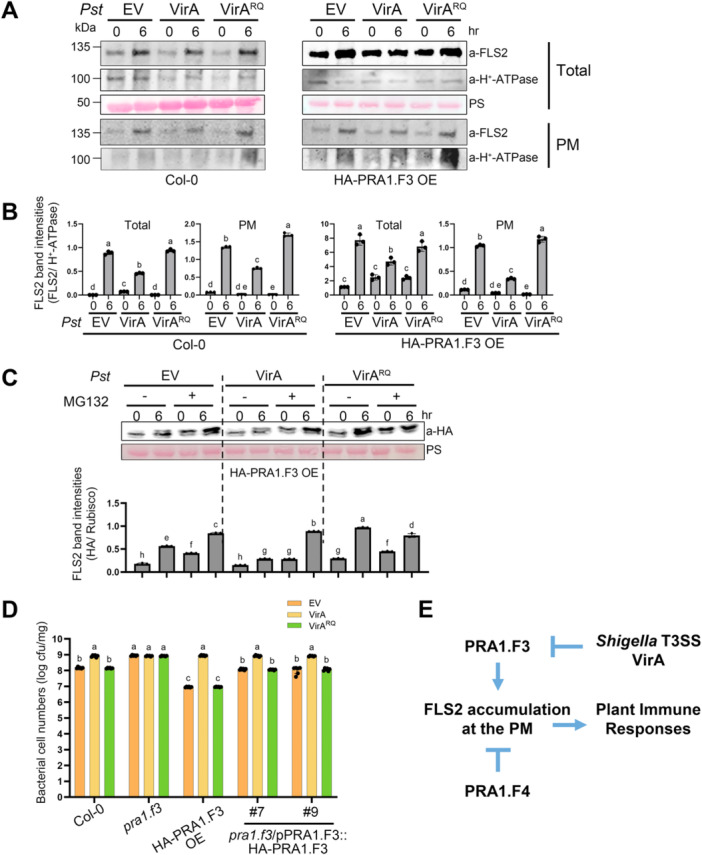
VirA‐mediated suppression of PRA1.F3 compromises immunity *in planta*. (A) VirA reduces endogenous FLS2 abundance during *Pst* infection. WT (Col‐0) and HA‐PRA1.F3 OE *Arabidopsis* seedlings were flood‐inoculated with *Pst* carrying an EV, VirA‐Flag, or the GAP‐inactive mutant VirA^RQ^‐Flag at 5 × 10⁵ cfu mL⁻¹. Total protein extracts and PM fractions were prepared, and FLS2 protein levels were analyzed by immunoblotting using an anti‐FLS2 antibody. (B) Quantification of VirA‐induced FLS2 reduction. Relative FLS2 band intensities from the immunoblots shown in (A) were quantified using ImageJ and normalised to the PM marker H⁺‐ATPase. Data represent mean ± SD, and statistical significance was determined by one‐way ANOVA followed by Tukey's HSD test (*p* < 0.05). (C) Proteasome‐dependent degradation of PRA1.F3 by VirA in planta. *Arabidopsis* leaves flood‐inoculated with *Pst* VirA‐Flag were treated with 50 µM MG132, and PRA1.F3 protein levels were analyzed in total protein extracts at the indicated time points by immunoblotting using an anti‐HA antibody. Relative PRA1.F3 band intensities were quantified using ImageJ. Statistical significance was determined by one‐way ANOVA followed by Tukey's HSD test (*p* < 0.05). (D) Genetic evidence that PRA1.F3 is required for VirA‐mediated virulence. Col‐0, *pra1.f3*, HA‐PRA1.F3 OE, and *pra1.f3*/*pPRA1.F3::HA‐PRA1.F3* seedlings were flood‐inoculated with *Pst* carrying EV, VirA‐Flag, or VirA^RQ^‐Flag at 5 × 10⁵ cfu mL⁻¹. Bacterial populations were quantified at 3 dpi. Data represent mean ± SD (*n* = 4). Different letters indicate statistically significant differences as determined by two‐way ANOVA followed by Tukey's HSD test (*p* < 0.05). (E) Model for VirA‐mediated suppression of FLS2 accumulation at PM and plant immunity. PRA1.F3 promotes FLS2 accumulation at the PM, thereby enhancing immune signalling, whereas PRA1.F4 negatively regulates this pathway. The *Shigella* T3SS effector VirA targets PRA1.F3, leading to its destabilization and impaired FLS2 accumulation at the PM, ultimately attenuating plant immune responses. [Color figure can be viewed at wileyonlinelibrary.com]

To determine whether VirA suppresses immunity through PRA1.F3 under native conditions, we monitored FLS2 and PRA1.F3 abundance in *A. thaliana* during infection. Consistent with the results obtained in *N. benthamiana*, expression of VirA by *Pst* VirA reduced PM‐localised FLS2 in both Col‐0 and HA–PRA1.F3 OE transgenic plants, whereas infection with the *Pst* VirA^RQ^ strain had little effect compared with the *Pst* EV (Figure 7A). Although HA–PRA1.F3 OE plants exhibited higher basal levels of FLS2 in both total protein extracts and PM fractions relative to Col‐0, the magnitude and trend of VirA‐induced FLS2 reduction were comparable between the two genotypes. Densitometric quantification of FLS2 protein levels from these immunoblots, normalised to the H^+^‐ATPase band intensity, is shown in Figure 7B. PRA1.F3 abundance was also decreased upon *Pst* VirA infection, and this reduction was reversed by MG132 treatment, supporting UPS‐mediated degradation of PRA1.F3 by VirA in planta (Figure 7C).

To assess whether VirA‐mediated reduction of FLS2 occurs at the transcriptional level, we examined *FLS2* transcript abundance in Col‐0 plants following infection with *Pst* EV, *Pst* VirA, or the *Pst* VirA^RQ^ strain. qRT‐PCR analysis revealed that *FLS2* transcript levels were not significantly altered by *Pst* VirA compared with the *Pst* EV control (Figure S12). In contrast, expression of FLS2 downstream defence marker genes, including *WRKY29*, *FRK1*, and *PR1*, was significantly suppressed upon *Pst* VirA infection (Figure S12). Notably, plants infected with *Pst* VirA^RQ^ exhibited expression patterns comparable to those observed upon *Pst* EV infection, indicating that suppression of FLS2‐dependent immune signalling requires VirA GAP activity and occurs without changes in *FLS2* transcription.

To genetically validate PRA1.F3 as a functional VirA target, we complemented the *pra1.f3* with a native promoter‐driven HA‐PRA1.F3 construct (Figure S13). This complementation restored VirA‐dependent susceptibility (Figure 7D), confirming that endogenous PRA1.F3 is required for VirA‐mediated immune suppression. Pathogenicity assays further showed that *Pst* VirA enhanced bacterial growth in Col‐0 but not with GAP‐inactive VirA^RQ^, while *pra1.f3* remained highly susceptible regardless of effector genotype. HAPRA1.F3 OE plants conferred enhanced resistance only in the absence of functional VirA. Notably, although HA‐PRA1.F3 OE lines restricted *Shigella* expressing VirA (Figure 3A), this resistance phenotype was abolished upon *Pst* VirA infection (Figure 7D), likely reflecting differences in effector delivery efficiency or timing between the two bacterial systems. Collectively, these results demonstrate that VirA represses PRA1.F3 via its GAP activity in planta, leading to destabilization of FLS2 and compromised immune competence.

## 4 Discussion

Pathogens have evolved diverse strategies to subvert plant immunity, among which the manipulation of vesicle trafficking pathways has emerged as a central mechanism (Yuen et al. 2023). In this study, we identified the *Shigella* T3SS effector VirA as a potent inhibitor of immune receptor stability at the PM in *Arabidopsis*. VirA specifically targets PRA1.F3, a trafficking regulator required for efficient PM localisation of the PRR FLS2. VirA‐mediated repression of PRA1.F3 results in reduced FLS2 accumulation and impaired immune signalling, revealing a previously unrecognised immune evasion strategy in which a bacterial effector dismantles host receptor stability by subverting endomembrane trafficking (Figure 7E).

Although FLS2 and EFR share similar domain architectures, signalling outputs, and ligand‐induced trafficking routes, our results demonstrate that PRA1.F3 differentially regulates these two receptors (Figures 4C and S9). While both receptors undergo ligand‐induced activation and endocytosis (Chinchilla et al. 2007; Nekrasov et al. 2009; Beck et al. 2012), their steadystate localisation and trafficking dependencies differ. FLS2 is predominantly maintained at the PM and requires continuous endomembrane‐mediated delivery to sustain immune competence, whereas EFR relies more strongly on ER‐associated regulatory pathways (Beck et al. 2012; Bücherl et al. 2017). Consequently, disruption of PRA1.F3‐dependent trafficking disproportionately affects FLS2, whose immune output critically depends on PM residence. Nevertheless, we do not exclude the possibility that PRA1.F‐dependent regulation extends to a subset of immune receptors sharing similar trafficking or localisation features with FLS2.

Although several transient expression assays in this study examined steady‐state, nonactivated FLS2, our infection assays address FLS2 regulation under physiologically relevant, ligand‐activated conditions (Figure 7A). Under basal conditions, FLS2 constitutively cycles between the PM and TGN/early endosome compartments, whereas perception of flg22 triggers rapid receptor internalisation that is essential for immune activation (Chinchilla et al. 2007; Beck et al. 2012). Because *Pst* delivers flg22 during infection, the VirA‐dependent reduction of endogenous FLS2 observed upon *Pst* VirA infection reflects an effect on activated receptor pools in planta. Notably, FLS2 abundance was significantly reduced under these conditions compared with *Pst* EV controls (Figure 7A), demonstrating that VirA suppresses FLS2 accumulation even during active immune signalling. While our data do not resolve the precise trafficking step affected following receptor activation, multiple lines of evidence argue that VirA primarily promotes receptor destabilization rather than selectively altering recycling dynamics. VirA reduces total FLS2 protein levels in a manner sensitive to MG132, induces UPS‐dependent degradation of PRA1.F3, and compromises a trafficking pathway required for efficient delivery and maintenance of FLS2 at the PM. Although indirect effects on flg22‐induced internalisation or post‐endocytic sorting cannot be excluded, the available data most consistently support a model in which VirA attenuates immune signalling by destabilising PRA1.F3, thereby reducing both steady‐state and activated pools of FLS2 at the PM.

Unlike animals and yeast, which encode only a few PRA1 genes, *Arabidopsis* possesses 19 PRA1 isoforms (Alvim Kamei et al. 2008), most of which remain poorly characterised. PRA1.B6 negatively regulates ER‐to‐Golgi trafficking and undergoes UPS‐mediated degradation (Lee et al. 2011), while PRA1.F4 localises to the Golgi and restricts protein targeting to the PM without affecting apoplastic secretion (Lee et al. 2017). Consistent with these reports, we found that PRA1.F4 preferentially localises to the Golgi and negatively regulates FLS2 trafficking and immune signalling. Notably, although PRA1.F3 and PRA1.F4 share similar biochemical properties, they display distinct subcellular localisations, suggesting that spatial compartmentalisation rather than intrinsic biochemical activity may be a key determinant of PRA1 isoform function. In line with this idea, a tomato PRA1 homologue related to PRA1.F4 promotes vacuolar targeting of RLP‐type PRRs via the TGN/EE pathway (Pizarro et al. 2018), highlighting the functional plasticity of this trafficking regulator family, depending on the species or specific pathogen context.

VirA functions as a Rab1 GAP in mammalian systems, disrupting ER‐to‐Golgi trafficking (Dong et al. 2012), and our data demonstrate that this virulence activity is conserved in plants. Although no Rab proteins were identified in our Y2H screen, VirA associates with Rab5 and Rab7 in planta in a C34‐dependent manner (Figure S14), likely through PRA1 scaffolds. PRA1 proteins recruit prenylated Rabs to specific endomembrane compartments (Alvim Kamei et al. 2008), and VirA localises to multiple trafficking hubs including the ER, the Golgi, and endosomes (Figure S3C). We propose that these PRA1‐Rab complexes form membrane‐associated platforms that facilitate access of VirA to vesicle trafficking pathways, thereby enabling selective disruption of immune receptor delivery and signalling during infection.

While our data support a model in which VirA suppresses immunity primarily by promoting UPS‐dependent degradation of PRA1.F3 and subsequent destabilization of FLS2, the precise trafficking step affected remains unresolved. Whether VirA interferes with receptor delivery, recycling, or post‐endocytic stability will require further investigation. In addition, VirA has been reported to destabilise microtubules in mammalian cells (Yoshida et al. 2006), raising the possibility that indirect effects on the cytoskeleton may further influence membrane trafficking in plants, an avenue for future study.

In conclusion, we identify PRA1.F3 as a key host target of the *Shigella* effector VirA and uncover a conserved mechanism by which a bacterial effector suppresses immune signalling by disrupting endomembrane trafficking. Our findings highlight functional specialisation among PRA1 isoforms and establish PRA1.F3 as a central vulnerability exploited by pathogens. Beyond its relevance to plant immunity, this work underscores the utility of plant systems for dissecting conserved virulence mechanisms across kingdoms.

## Supporting information

Supporting File
