# Supplementary material for: Shigella Effector VirA Suppresses Plant Immunity by Compromising PRA1.F3‐Dependent Accumulation of FLS2 at the Plasma Membrane
Source: Plant Cell Environ. 2026 Apr 14;49(8):5267–82. doi: 10.1111/pce.70541 (PMC13353661; doi:10.1111/pce.70541)
Supplement: Supplementary file 1 — Supporting File [file PCE-49-5267-s001.docx]

**Supporting information**

Jo et al.

**Table S1.** List of key reagents and materials used in this study.

| Reagent or Resource | Source | Identifier |
| --- | --- | --- |
| Antibodies | | |
| Anti-FLAG | Cell Signaling Technology | Cat #2368; RRID:AB_2217020 |
| Anti-GFP | Santa Cruz | Cat #sc-9996; RRID:AB_627695 |
| Anti-HA | Sigma | Cat #H9658; RRID:AB_ 260092 |
| Anti-phospho-p44/p42 MAPK (ERK1/2) | Cell Signaling Technology | Cat #4377; RRID:AB_331775 |
| Anti-mCherry | BioRad | Cat #AHP2326 |
| Anti-ubiquitin | Santa Cruz | Cat #sc-8017 RRID:AB_628423 |
| Anti-H^+^-ATPase | Agrisera | Cat #AS07260 RRID:AB_1031584 |
| Anti-FLS2 | Agrisera | Cat #AS121857 |
| Bacterial strains | | |
| *Shigella flexneri* 5a M90T | Onodera et al. | N/A |
| *Shigella flexneri* 5a M90T Δ*virA* | This study | N/A |
| *Shigella flexneri* 5a M90T Δ*virA +*pVirA | This study | N/A |
| *Shigella flexneri* 5a BS176 | Sansonetti et al. | N/A |
| *Pseudomonas syringe pv. tomato* DC3000 | Buell et al. | N/A |
| *Pseudomonas syringe pv. tomato* DC3000 GFP | Wang et al. | N/A |
| *Pseudomonas syringe pv.tomato* AvrRpt2 | Whalen et al. | N/A |
| *Agrobacterium tumefaciens* GV2260 | Deblaere et al. | N/A |
| Chemicals, peptides, and recombinant proteins | | |
| GFP-Trap Agarose | Chromo Tek | Cat #gta-20 |
| Pierce Anti-HA Magnetic beads | Termo scientific | Cat #88836 |
| Protease Inhibitor Cocktail Tablets | Roche | Cat #04693116001 |
| flg22 peptide | Alpha Diagnostics, Inc | Cat #FLG22-P-1 |
| MG132 | Sigma-Aldrich | Cat #474790 |
| ConA | Sigma-Aldrich | Cat #C9705 |
| Critical commercial assays | | |
| Gateway™ BP Clonase™ II Enzyme mix | Invitrogen | Cat #11789020 |
| Gateway™ LR Clonase™ II Enzyme mix | Invitrogen | Cat #11791020 |
| NucleoSpin RNA Plant | MN | Cat #740949.50 |
| M-MLV reverse transcriptase | Invitrogen | Cat #28025013 |
| iQ™ SYBR® Green Supermix | BioRad | Cat ##1708880 |
| Minute Plasma Membrane Protein Isolation Kit for Plants | Invent Biotechnologies | Cat #SM-005-P |
| Experimental models: Organisms/strains | | |
| Arabidopsis: Col-0 | ABRC | CS22625 |
| Arabidopsis*: pra1.b5* | ABRC | SALK_043145 |
| Arabidopsis*: pra1.e* | ABRC | SAIL_1307_G02 |
| Arabidopsis*: pra1.f3* | ABRC | SALK_079876 |
| Arabidopsis*:pra1.f4* | Lee et al. | N/A |
| Arabidopsis*:pra1.g1* | ABRC | GK-292C11 |
| Arabidopsis*:* HA-PRA1.B5 OE | This paper | N/A |
| Arabidopsis: HA-PRA1.E OE | This paper | N/A |
| Arabidopsis*:* HA-PRA1.F3 OE | This paper | N/A |
| Arabidopsis*:* HA-PRA1.F4 OE | This paper | N/A |
| Arabidopsis*:* HA-PRA1.G1 OE | This paper | N/A |
| Arabidopsis*: pra1.f3/*pPRA1.F3::HA-PRA1.F3 | This paper | N/A |
| Recombinant DNA | | |
| pBAV179-ViA-HA | This paper | N/A |
| pBAV179-ViA^C34S^-HA | This paper | N/A |
| pBAV179-ViA^R188K^-HA | This paper | N/A |
| pBAV179-ViA^Q280A^-HA | This paper | N/A |
| pBAV179-ViA^RQ^-HA | This paper | N/A |
| pBAV179-ViA-Flag | This paper | N/A |
| pBAV179-ViA^RQ^-Flag | This paper | N/A |
| pBAV178-ViA-AvrRpt2^101-265^ | This paper | N/A |
| pGWB11-35S::PRA1.E-FLAG | This paper | N/A |
| pk7FWG2-35S::VirA-GFP | This paper | N/A |
| pk7FWG2-35S::VirA^C34S^-GFP | This paper | N/A |
| pk7FWG2-35S::VirA^R188K^-GFP | This paper | N/A |
| pk7FWG2-35S::VirA^Q280A^-GFP | This paper | N/A |
| pk7FWG2-35S::VirA^RQ^-GFP | This paper | N/A |
| pSPYNE-35S::PRA1.E-YFP^N^ | This paper | N/A |
| pSPYCE-35S::PRA1.E-YFP^C^ | This paper | N/A |
| pSPYNE-35S::VirA-YFP^N^ | This paper | N/A |
| pSPYCE-35S::VirA-YFP^C^ | This paper | N/A |
| pSPYNE-35S::VirA^C34S^-YFP^C^ | This paper | N/A |
| pSPYCE-35S::VirA^C34S^-YFP^C^ | This paper | N/A |
| pBIB-35S::HA-PRA1.E | This paper | N/A |
| pBIB-35S::HA-PRA1.F3 | This paper | N/A |
| pBIB-35S::HA-PRA1.F4 | This paper | N/A |
| pK2GW7K35S::ST-GFP | This paper | N/A |
| pK7WGY2-35S::YFP-ARA5 | This paper | N/A |
| pK7YWG2-35S::ARA6-YFP | This paper | N/A |
| pGWB455-35S::mRFP-PRA1.F3 | This paper | N/A |
| pGWB455-35S::mRFP-PRA1.F4 | This paper | N/A |
| FLS2prom::FLS2-3xmyc-GFP | Robatzek et al. | Addgene Plasmid #86157 |
| FLS2p::FLS2-3xmyc-mCherry | Mbengue et al. | Addgene Plasmid #86159 |
| AtUBQ10::mCherry-MtRAB5A2 | Sergey Ivanov and Maria J. Harri. | Addgene Plasmid #61185 |
| AtUBQ10::GMMAN49-mCherry | Sergey Ivanov and Maria J. Harri. | Addgene Plasmid #61172 |
| AtUBQ10::AtWAK2sp-mCherry-HDEL | Sergey Ivanov and Maria J. Harri. | Addgene Plasmid #61170 |
| AtUBQ10:AtPIP2a-mCherry | Sergey Ivanov and Maria J. Harri. | Addgene Plasmid #61180 |
| 35S::EFR WT-mgfp5 | Robatzek et al. | Addgene plasmid # 102413 |
| Software and algorithms | | |
| NIS-Elements AR | Nikon Microscope Products | <https://www.microscope.healthcare.nikon.com/> |
| Prism 8 | GraphPad | <https://www.graphpad.com/> |
| SPSS v.18 | IBM | <https://www.ibm.com/kr-ko/spss> |
| ImageJ | GitHub | <https://github.com/imagej/ImageJ> |

**Table S2**. Primer sequences used in this study.

| **Primer list** | **Nucleotide sequence** |
| --- | --- |
| attB F | 5′-GGGGACAAGTTTgtacaaaaaagcaggc tTC-3′ |
| attb R | 5′-GGGGACCACTTTGTACAAGaaagctgg gtc-3′ |
| attB VirA-F | 5′-AAAGCAGGCTTCGCATTAATAGGAAAATAC-3′ |
| attB VirA- R | 5′-GAAAGCTGGGTCAACATCAGGAGATATGAT-3′ |
| attB PRA1.E-F (Flag) | 5′-AAAGCAGGCTTCATGAATCAGAAACCTCCG-3′ |
| attB PRA1.E-R (Flag) | 5′-GAAAGCTGGGTCAATTGGGGTATAACTCGA-3′ |
| attB PRA1.F3-F (RFP) | 5′-AAAGCAGGCTTCATGACGAACTACGGT-3′ |
| attB PRA1.F3-R (RFP) | 5′-GAAAGCTGGGTCTTAAGTCGACGGGTATGA-3′ |
| attB PRA1-F4-F (RFP) | 5′-AAAGCAGGCTTCATGGCGAACAACGACGAG-3′ |
| attB PRA1.F4-R (RFP) | 5′-GAAAGCTGGGTCTTAAGACGAAGGGTGTGA-3′ |
| PRA1.B5-F (HA) | 5′-CGCGGATCCGAATGGTGTCAACAAATCCT-3′ |
| PRA1.B5-R (HA) | 5′-CCGCTCGAGTTATTACACGGGGGAAGC-3′ |
| PRA1.E-F (HA) | 5′-CGCGGATCCGAATGAATCAGAAACCTCCG-3′ |
| PRA1.E-R (HA) | 5′-CCGCTCGAGTTATCAAATTGGGGTATA-3′ |
| PRA1.F3-F (HA) | 5′-CGCGGATCCGAATGACGAACTACGGTGCGATT-3′ |
| PRA1.F3-R (HA) | 5′-CCGCTCGAGTTATTAAGTCGACGGGTA-3′ |
| PRA1.F4-F (HA) | 5′-CGCGGATCCGAATGGCGAACAACGACGAGATT-3′ |
| PRA1.F4-R (HA) | 5′-CCGCTCGAGTTAAGACGAAGGGTGTGA-3′ |
| PRA1.G1-F (HA) | 5′-CGCGGATCCGAATGTTAGCACCGGGGGAG-3′ |
| PRA1.G2-R (HA) | 5′-CCGCTCGAGTTATCAGCTCCAAATGAAG-3′ |
| 16S- F (Q-PCR) | 5′-GCGGTTTGTTAAGTCAGATGTGAAA-3' |
| 16S- R (Q-PCR) | 5'-GACTCAAGCTTGCCAGTATCAGAT-3' |
| VirA-F (Q-PCR) | 5′-AACTTCCCCCAACAAGACCT-3′ |
| VirA-R (Q-PCR) | 5′-GGGACAACTGCGTTGATTTT-3′ |
| FLS2-F (Q-PCR) | 5′-GCGAAACAGAGCTTTGAACC-3′ |
| FLS2-R (Q-PCR) | 5′-GTGTCGTAACGAACCGATGA-3′ |
| PRA1.F3-F (Q-PCR) | 5′-CGGACGGTCTTGATTGTTTT-3′ |
| PRA1.F3-R (Q-PCR) | 5′-GACGTCAGCCCAGAAGTCTC-3′ |
| PRA1.F4-F (Q-PCR) | 5′-CTGGAGGGTGATGTTCGATT-3′ |
| PRA1.F4-R (Q-PCR) | 5′-CCAAACCGGTAAAGACGATG-3′ |
| Actin-F (Q-PCR) | 5′-ACCACAACAGCAGAGCGGGA-3′ |
| Actin-R (Q-PCR) | 5′-CCAATCGTGATGACTTGCCCA-3′ |
| WRKY29-F (Q-PCR) | 5′-CCCGGAGAAATTCACCATAA-3′ |
| WRKY29-R (Q-PCR) | 5′-ATCAGCGGATGGGATCATAG-3′ |
| FRK1-F (Q-PCR) | 5′-TATCTTGAGCTGGGAAGAGAGG-3′ |
| FRK1-R (Q-PCR) | 5′-AGTCGAATAGTACTCGGGGTCA-3′ |
| PR1-F (Q-PCR) | 5′-CGTTCACATAATTCCCACGAG-3′ |
| PR1-R (Q-PCR) | 5′-TCAGTGAGACTCGGATGTGC-3′ |

**Table S3.** Summary of candidate VirA-interacting proteins identified from the Y2H screening

| **Identified pray** | **Reporter expression** | **In planta Co-IP with VirA** | **Accession No.** | **Description** |
| --- | --- | --- | --- | --- |
|  | ***HIS3 ADE2*** |  |  |  |
| **PRA1.E** | + | Positive | NM_100751 | *A. thaliana* prenylated RAB acceptor 1.E |
|  | + |  |  |  |
| **BPA1** | + | Not detected | NM_121690 | *A.thaliana* binding partner of acd11 1 |
|  | + |  |  |  |
| **aoc1** | + | Not detected | AJ308483 | *A. thaliana* mRNA for allene oxide cyclase |
|  | + |  |  |  |
| **TIM17-2** | + | Not detected | NM_129296 | *A. thaliana* translocase inner membrane subunit 17-2 |
|  | + |  |  |  |
| **ALB1** | + | Not detected | NM_100725 | *A. thaliana* ALBINA 1 |
|  | + |  |  |  |


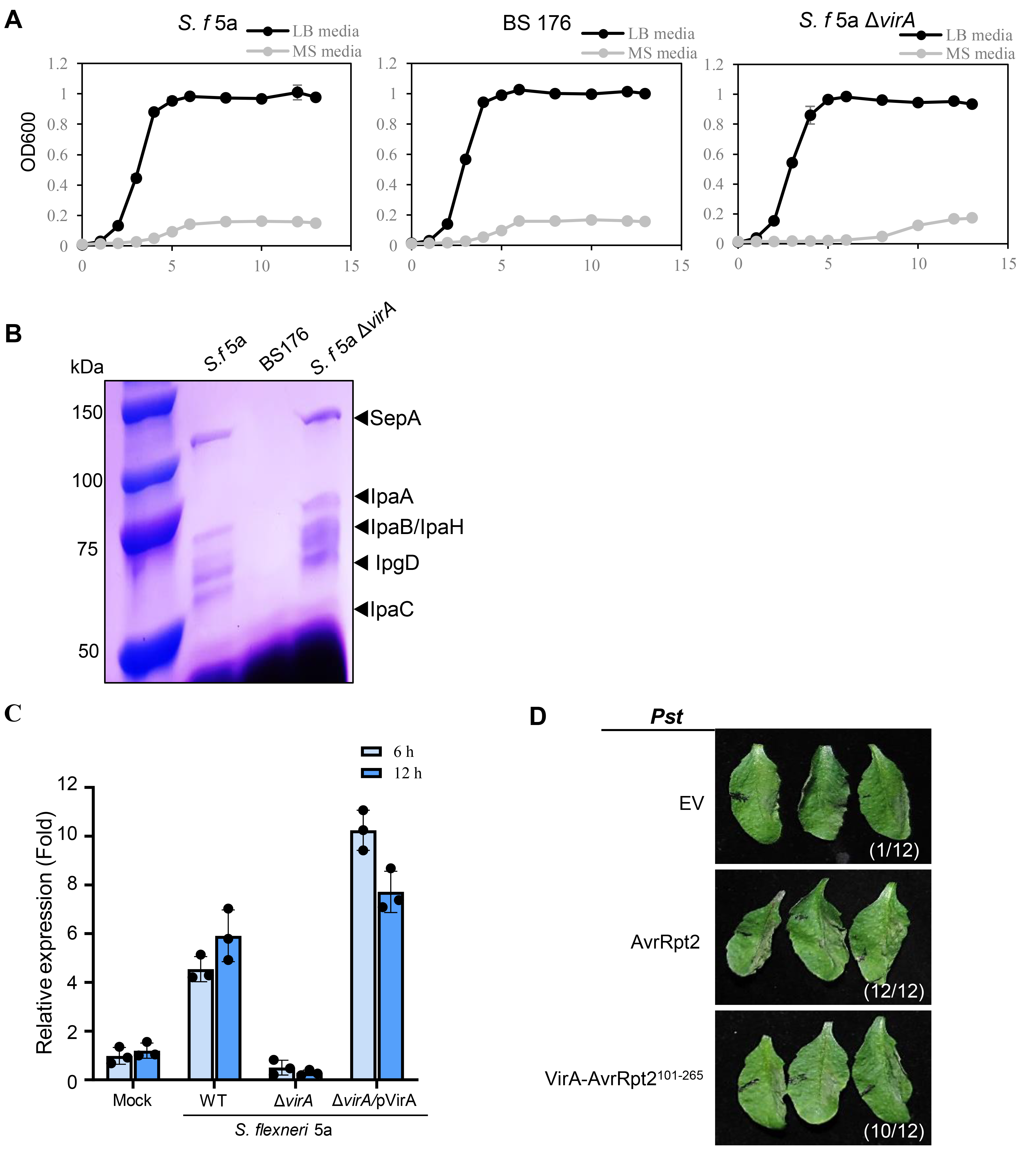


**Figure S1. Deletion of *virA* does not affect bacterial growth or T3SS-mediated effector secretion in *Shigella*. (A)** Growth of WT *S. flexneri* 5a, its T3SS-deficient mutant BS176, and Δ*virA* mutant on LB and MS media. **(B)** T3SS-dependent effector secretion in WT *S. flexneri* 5a, BS176, and ∆*virA* strains. Bacteria were cultured in tryptic soy broth at 37°C for 3 h in the presence of Congo red. Secreted proteins in the culture supernatants were separated by SDS-PAGE and visualized with Coomassie Brilliant Blue R staining. Protein size markers (kDa) are shown on the left and major effector proteins are indicated on the right. **(C)** *VirA* expression during *Arabidopsis* infection*.* Two-week-old seedlings were flood-inoculated with S. flexneri 5a WT, ΔvirA, or ΔvirA complemented with pVirA at 5 × 10⁵ cfu/mL. *VirA* transcript levels were measured by qRT-PCR at 6 and 12 hpi in infected leaves and normalized to *16S* *rRNA* (n = 9). (**D**) Translocation of VirA into plant cells via a heterologous T3SS delivery system. *Arabidopsis* leaves were infiltrated with Pst expressing EV, AvrRpt2*,* or a VirA-AvrRpt2¹⁰¹–²⁵⁵ fusion. The HR was visually assessed at 1 dpi. Numbers indicate the ratio of HR-positive leaves to total leaves examined.


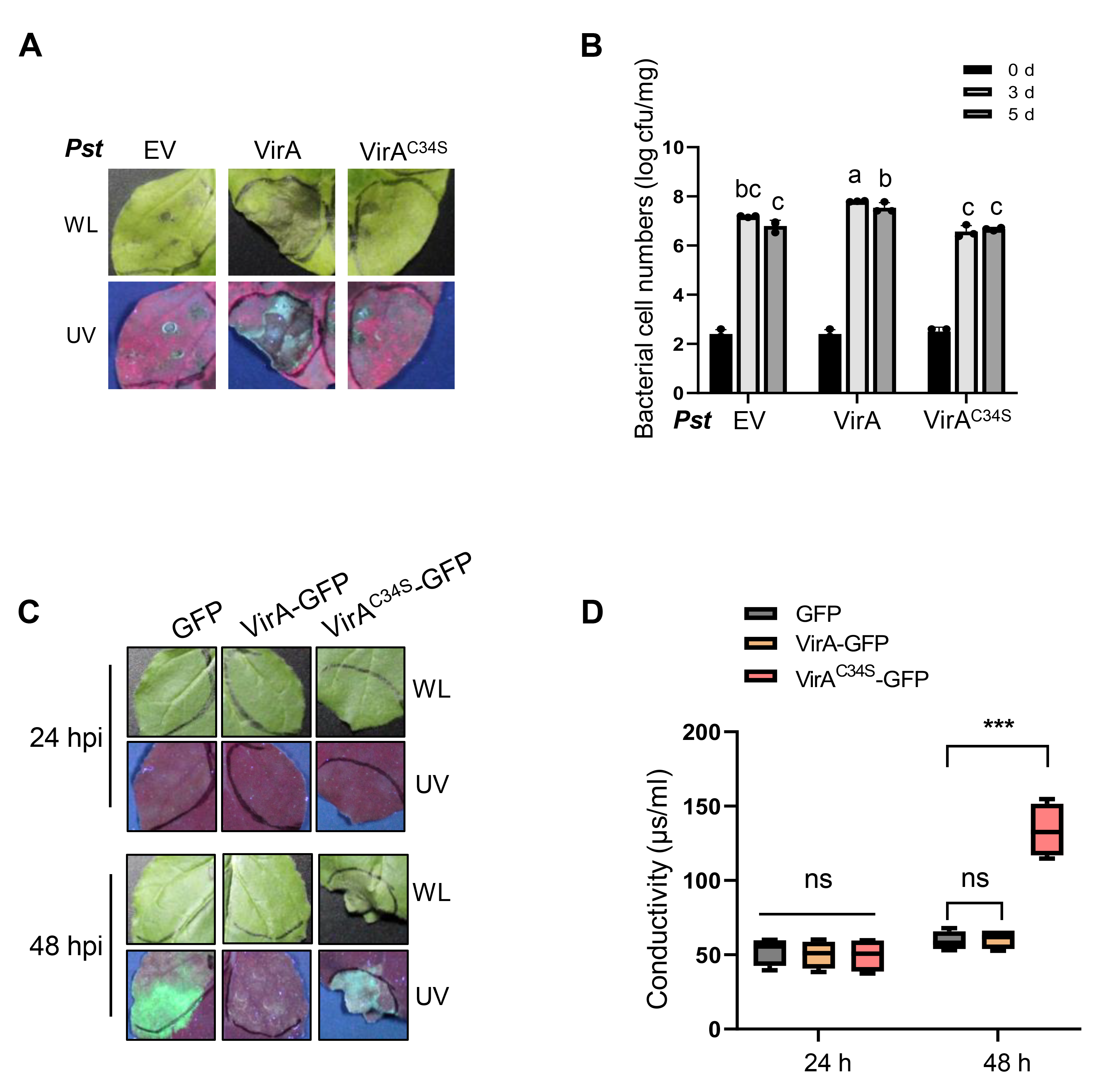


**Figure S2. Avirulence activity and HR-like cell death triggered by VirA^C34S^ in *N. benthamiana.* (A, B)** Comparison of in planta virulence of VirA and VirA^C34S^ in *N. benthamiana*. Leaves were syringe-infiltrated with *Pst* strains carrying an EV, VirA-HA, or VirA^C34S^-HA at 5 × 10⁵ cfu/ml. Representative symptoms were photographed at 5 dpi **(A)**, and bacterial populations were quantified at 0, 3, and 5 dpi (**B**). Data represent means ± SD (n = 3). Different letters indicate statistically significant differences as determined by a two-way ANOVA followed by Tukey’s HSD test (P < 0.05). **(C, D)** Expression of VirA^C34S^-GFP in *N. benthamiana* leaves triggered HR-like cell death, as shown by macroscopic tissue collapse **(C)** and electrolyte leakage **(D)**. In **(D)**, six leaf discs were excised from infiltrated areas, and ion leakage was measured at the indicated time points. Data represent mean ± SD (n = 4). Different letters indicate significant differences among samples, as assessed using a two-way ANOVA and Tukey's HSD (P < 0.05)

**
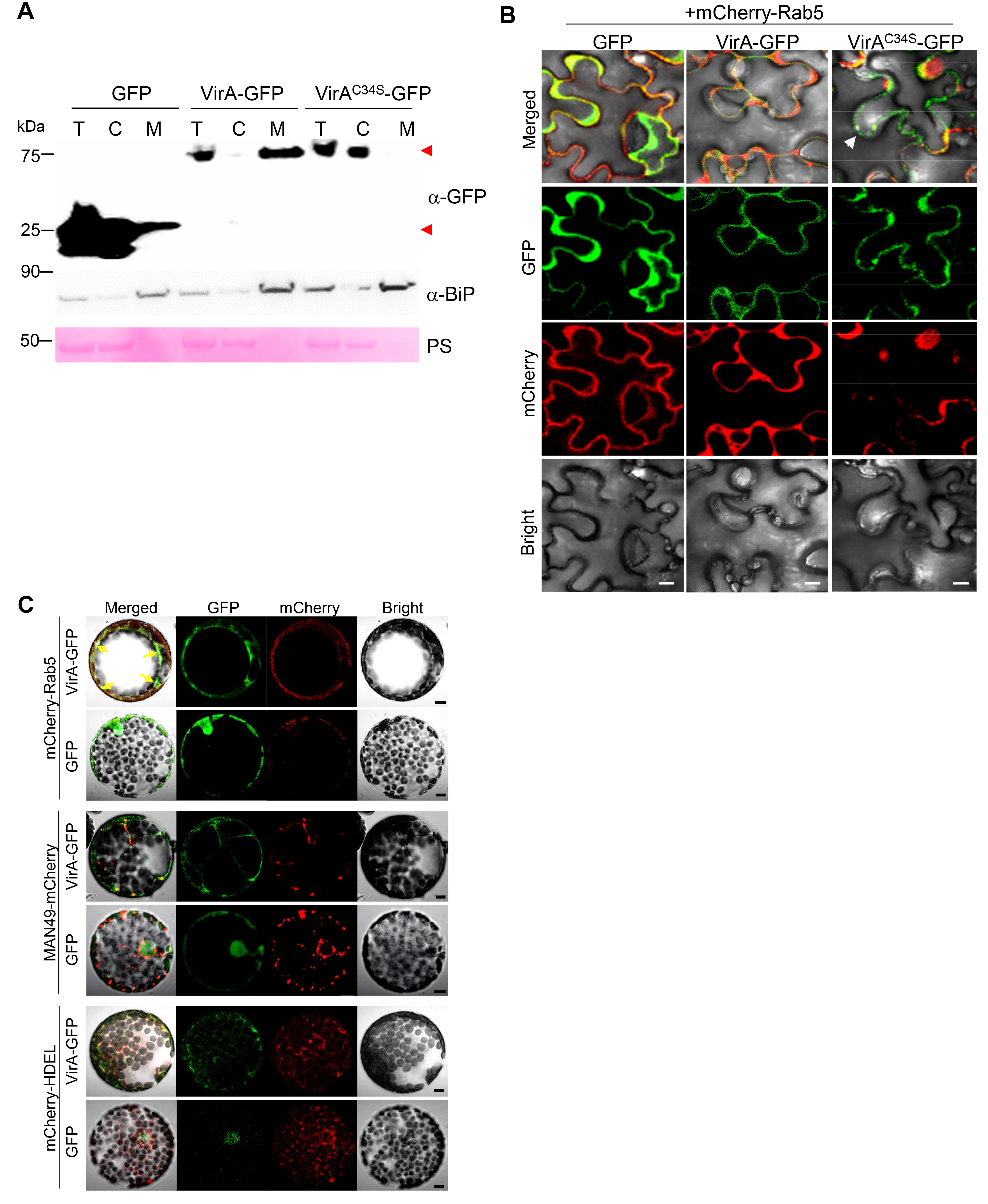
**

**Figure S3. Subcellular localization of VirA in endomembrane compartments. (A)** Subcellular fractionation of VirA-GFP and VirA^C34S^-GFP in *N. benthamiana*. Total protein were separated into membrane and soluble fractions at 1 dpi, followed by immunoblotting with indicated antibodies T, total protein fraction; C, cytosolic (soluble) fraction; M, membrane fraction. PM enrichment was verified using an anti–H⁺-ATPase antibody as a PM marker. PS staining of RuBisCO was used as a loading control. **(B)** Confocal microscopy of VirA^C34S^-GFP co-expressed with the early endosome marker mCherry-Rab5. VirA^C34S^-GFP showed largely diffuse cytosolic fluorescence with no strong colocalization with Rab5. Imaging was constrained by HR-like cell death induced by VirA^C34S^ expression. White arrowheads indicate regions of cytosolic signal and loss of membrane association. **(C)** VirA-GFP was coexpressed with mCherry-Rab5 (EE marker), MAN49-mCherry (Golgi marker), or mCherry-HDEL (ER marker) in *N. benthamiana* leaves via *Agrobacterium*-mediated infiltration. At 2 dpi, fluorescence signals were visualized in isolated protoplasts using confocal microscopy. Scale bars = 10 μm.


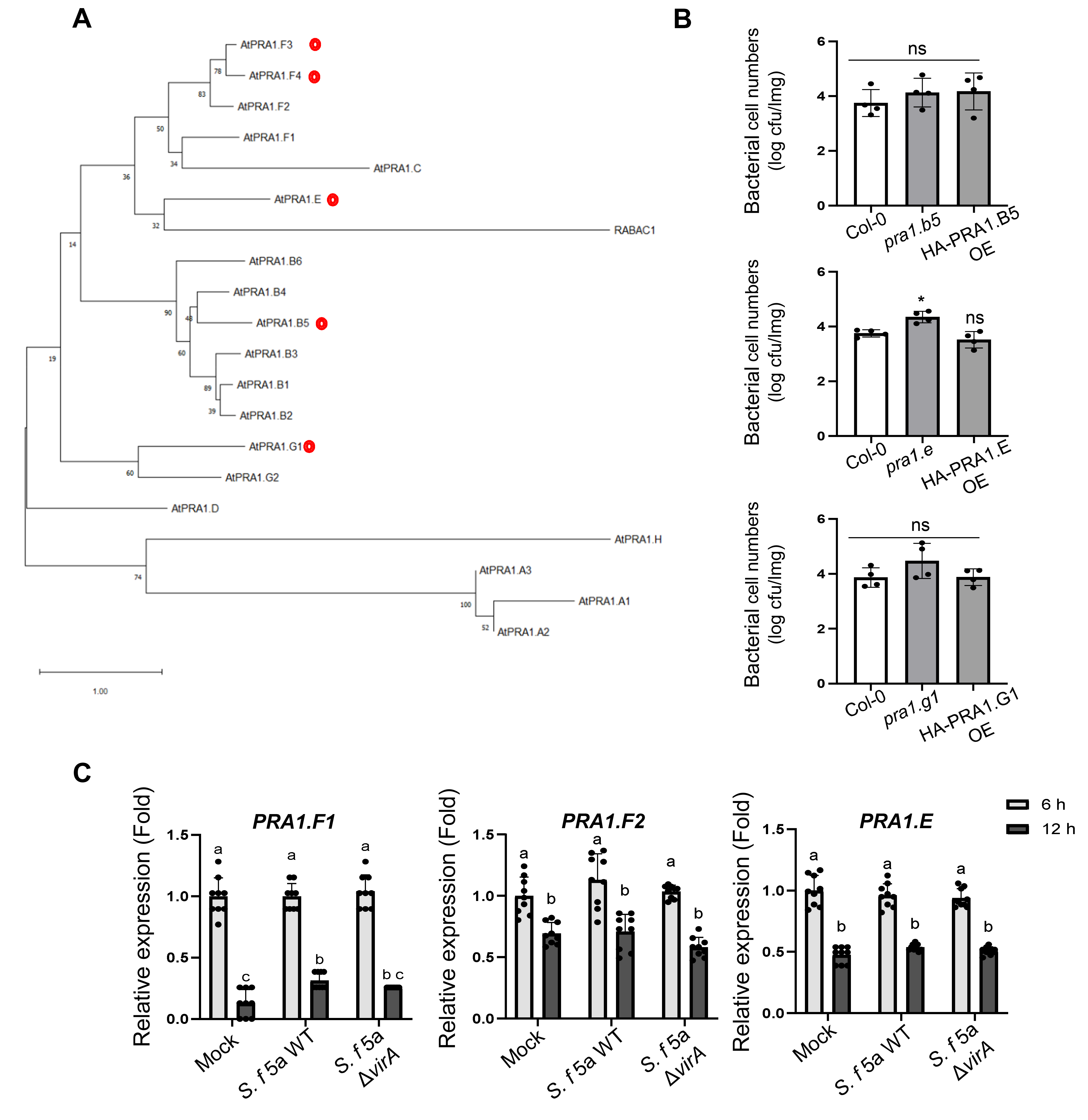


**Figure S4. Characterization of *Arabidopsis* PRA1 isoforms and their effects on *Shigella* infection*.* (A)** Phylogenetic analysis of Arabidopsis PRA1 family proteins. The phylogenetic tree was constructed using MEGA11 software with the Neighbor-Joining method. Bootstrap values (1,000 replicates) indicate the confidence level for each branch. The scale bar denotes branch length. PRA1 proteins marked with red circles were selected for further analysis. Human PRA1 was included in the phylogenetic analysis as an outgroup to provide evolutionary context. **(B)** Functional analysis of PRA1.B5, PRA1.E, and PRA1.G1 in *S. flexneri 5a* infection. WT (Col-0), T-DNA insertion mutants (*pra1.b5*, *pra1.e*, and *pra1.g1*), and OE lines (HA-PRA1.B5 OE, HA-PRA1.E OE, and HA-PRA1.G1 OE*)* were flood-inoculated with *S. flexneri* 5a, and bacterial proliferation was quantified at 3 dpi. Data represent means ± SD (n = 4). Different letters indicate statistically significant differences as determined by a one-way ANOVA followed by Tukey’s HSD test (P < 0.05). **(C)** Expression analysis of *PRA1* genes upon *S. flexneri* 5a infection*.* Arabidopsis seedlings were flood-inoculated with WT *S. flexneri* 5a or the ∆*virA* mutant strain at 5 × 10⁵ cfu/ml. Transcript levels of *PRA1.F1*, *PRA1.F2*, and *PRA1.E* were measured at 6 and 12 hpi by qRT-PCR. *AtActin* was used as an internal reference for normalization. Data represent means ± SD (n = 9). Different letters indicate statistically significant differences as determined by a two-way ANOVA followed by Tukey’s HSD test (P < 0.05).


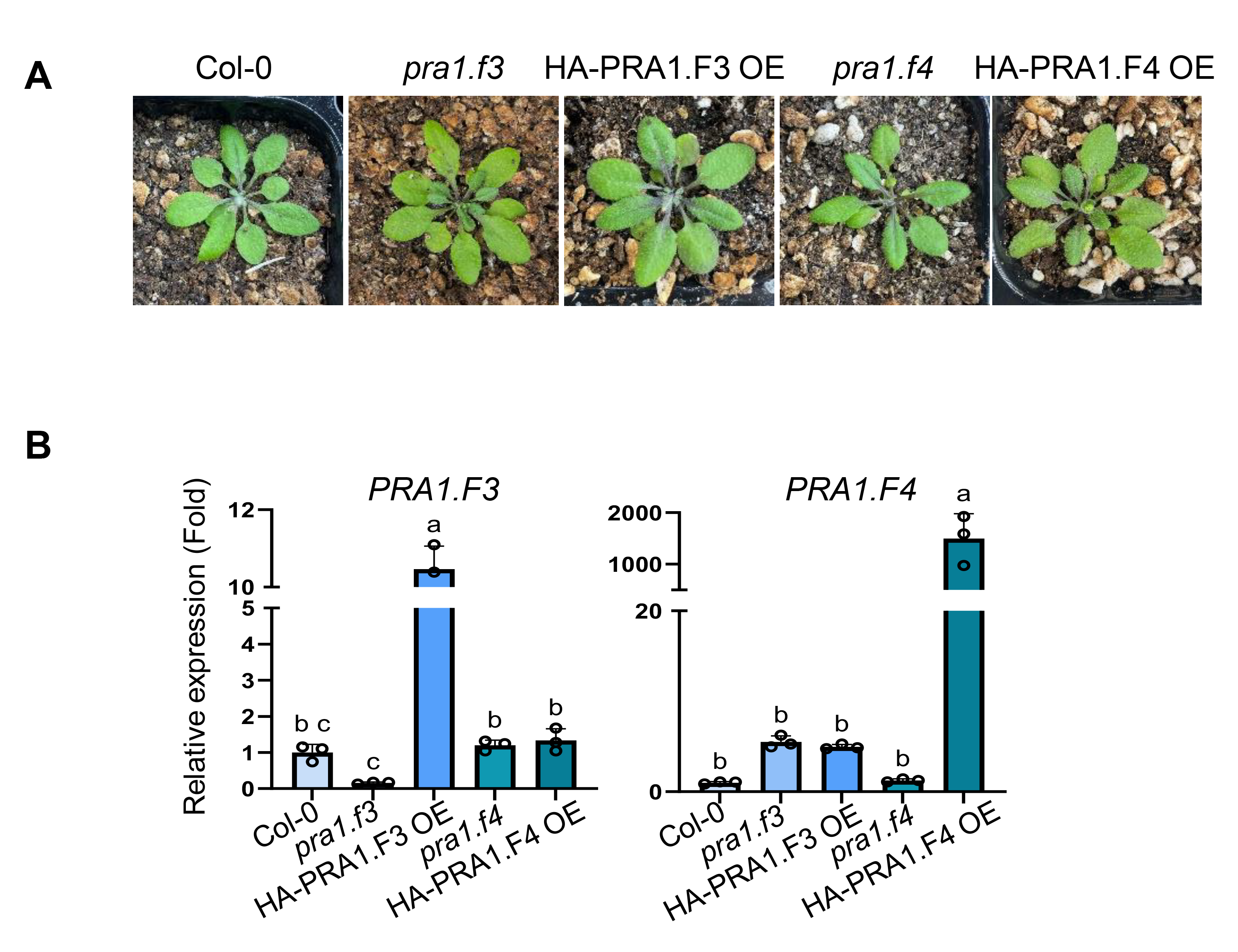


**Figure S5. Comparison of loss- and gain-of-function lines for Arabidopsis PRA1.F3 and PRA1.F4. (A)** Rosette phenotypes of 4-week-old soil-grown Arabidopsis Col-0, *pra1.f3* and *pra1.f4* mutants, and OE lines (HA-PRA1.F3 OE, and HA-PRA1.F4 OE). **(B)** Expression levels of *PRA1.F3* and *PRA1.F4* in mutant and OE lines determined by qRT-PCR. Transcript levels were normalized to *AtActin*. Data represent means ± SD (n = 3). Different letters indicate statistically significant differences as determined by a one-way ANOVA followed by Tukey’s HSD test (P < 0.05).

**
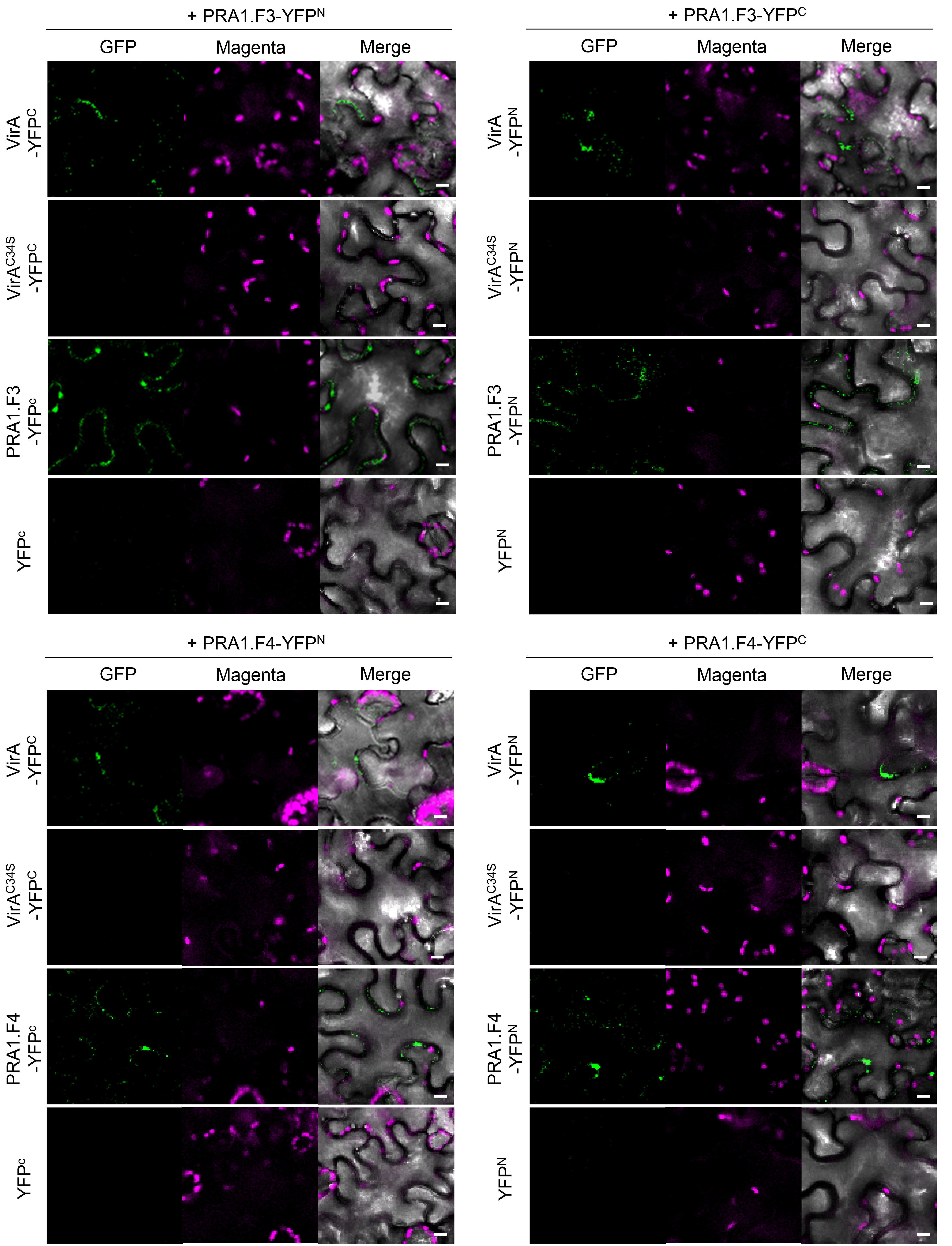
**

**Figure S6. BiFC analysis of VirA interactions with PRA1.F3 and PRA1.F4.** BiFC assays were performed in N. benthamiana epidermal cells to examine interactions between PRA1.F3 or PRA1.F4 and either WT VirA or VirA^C34S^. PRA1.F3 and PRA1.F4 were fused to the N- or C-terminal half of YFP, while VirA or VirA^C34S^ was fused to the complementary YFP fragment; both reciprocal fusion orientations were tested. Reconstituted YFP fluorescence indicates protein-protein interaction. Robust YFP signals were observed between WT VirA and PRA1.F3 or PRA1.F4 in both orientations, whereas no detectable signal was observed with VirA^C34S^, indicating that the conserved C34 residue is required for these interactions in planta. Confocal images were acquired at 2 dpi. Scale bars = 10 µm.


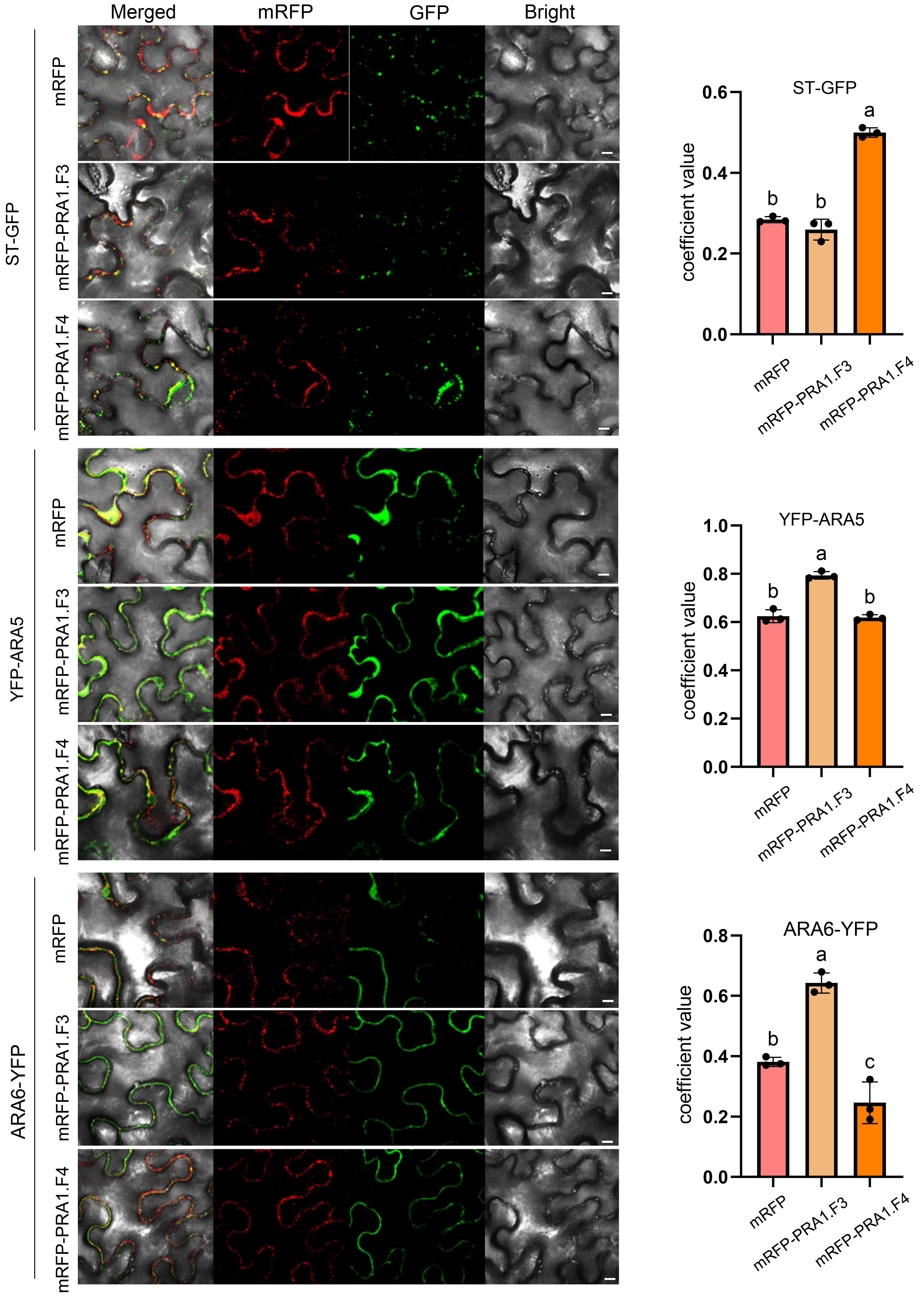


**Figure S7.** **Differential subcellular localization of PRA1.F3 and PRA1.F4 relative to endomembrane markers.** mRFP, mRFP-PRA1.F3, or mRFP-PRA1.F4 were co-expressed with ST-GFP (Golgi marker), YFP-ARA5, or ARA6-YFP (endosomal markers) in *N*. *benthamiana* epidermal cells. Confocal images were acquired at 2 dpi. Merged images show overlap between mRFP (red) and GFP/YFP (green) signals. Bright-field images are shown for cellular context. Scale bars = 10 µm. Colocalization was quantified using Pearson’s correlation coefficient from multiple independent cells. Bar graphs show mean ± SD. Different letters indicate statistically significant differences (one-way ANOVA with Tukey’s HSD, P < 0.05).


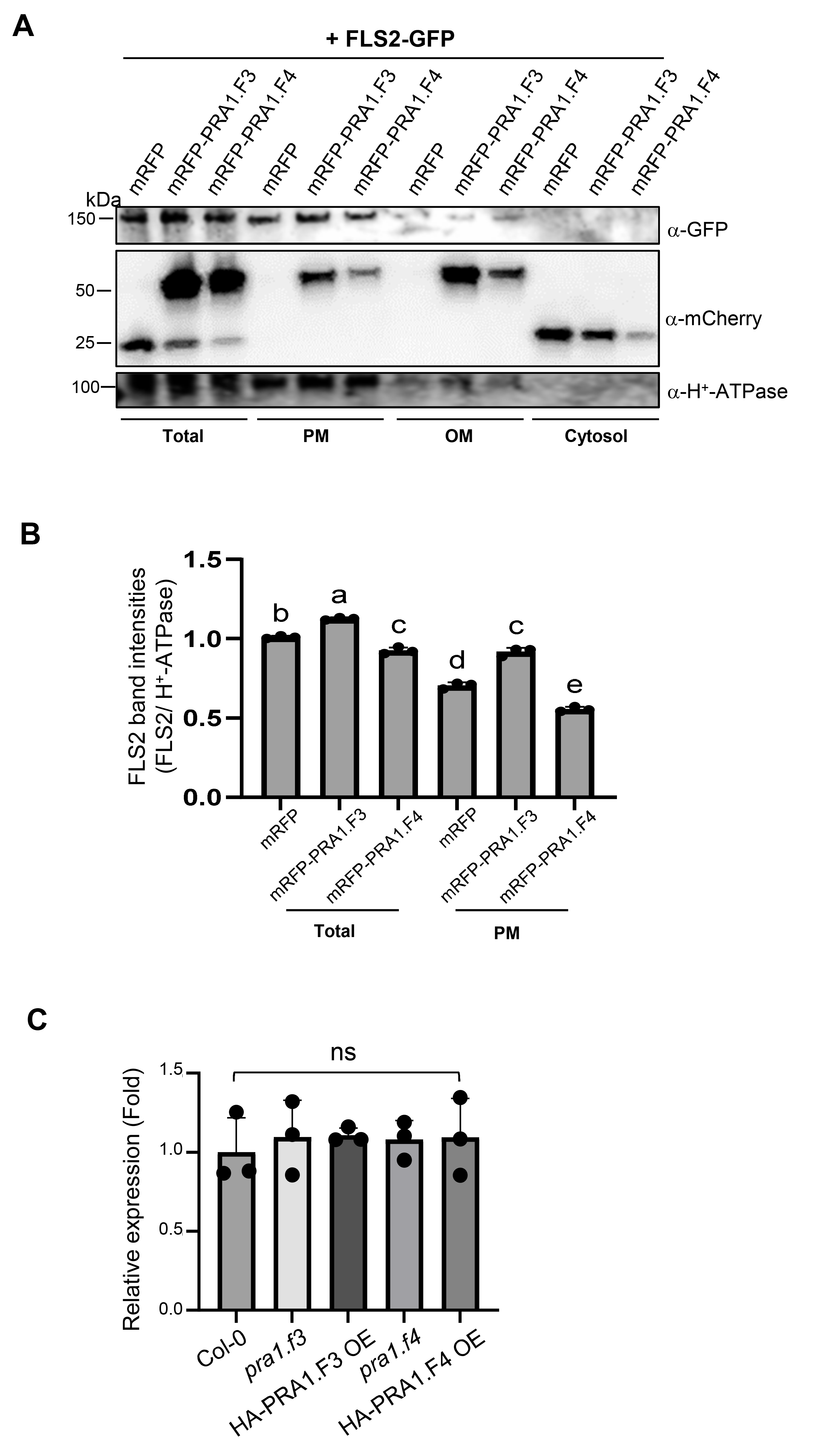


**Figure S8.** **PRA1.F3 and PRA1.F4 positively and negatively, respectively, regulate the trafficking of FLS2 to the PM. (A)** Effect of mRFP-PRA1.F3 and mRFP-PRA1.F4 on FLS2-GFP levels in the PM, organellar membrane (OM), and cytosol. Total protein extracts from *Agrobacterium*-infiltrated *N. benthamiana* leaves were separated into soluble and membrane fractions, and these fractions were analyzed by immunoblotting using anti-GFP, anti-mCherry, and anti-H+-ATPase antibodies. **(B)** Relative FLS2 band intensities in total and PM fractions were quantified from immunoblots shown in **(A)** using ImageJ and normalized to the plasma membrane marker H⁺-ATPase. Different letters indicate statistically significant differences (one-way ANOVA with Tukey’s HSD, P < 0.05). **(C)** qRT-PCR analysis of *FLS2* transcript levels in Arabidopsis Col-0, *pra1.f3, pra1.f4*, HA–PRA1.F3 OE, and HA–PRA1.F4 OE plants. Transcript levels were normalized to *AtActin*. Data represent means ± SD (n = 3). No statistically significant differences were detected according to Student’s t-test (P ≥ 0.05; ns, not significant).


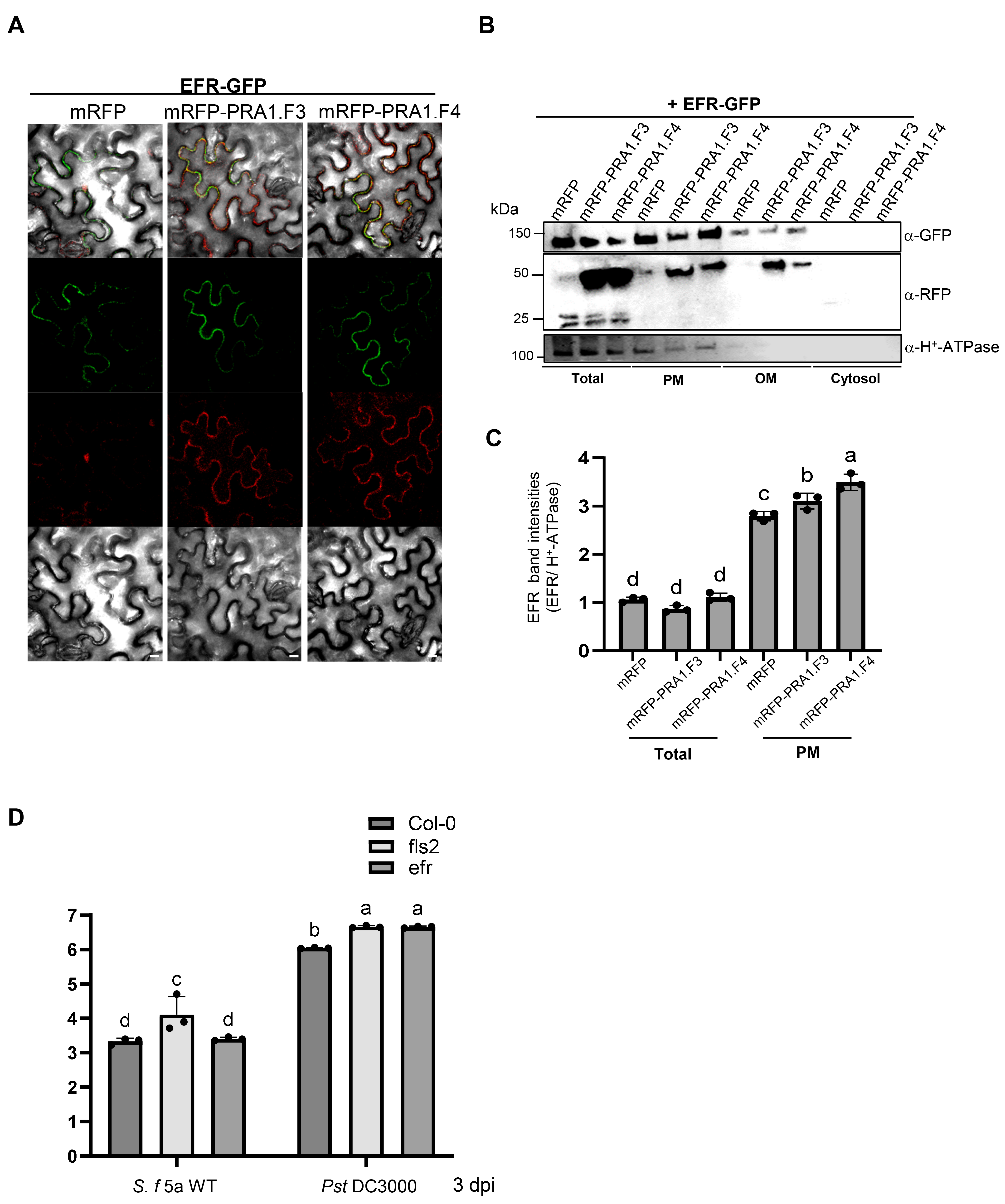


**Figure S9. PRA1.F3 and PRA1.F4 do not regulate EFR plasma membrane accumulation or EFR-dependent immunity. (A)** Subcellular localization of EFR-GFP in the presence of PRA1 isoforms. EFR-GFP was co-expressed with mRFP, mRFP-PRA1.F3, or mRFP-PRA1.F4 in *N. benthamiana* leaves. Confocal images were acquired at 2 dpi. Representative merged, GFP, mRFP, and bright-field images are shown. Scale bars = 10 µm. **(B)** Subcellular fractionation of EFR-GFP co-expressed with PRA1 isoforms. Total protein extracts were separated into total, PM, OM, and cytosolic fractions. EFR-GFP and PRA1 isoforms were detected by immunoblotting using anti-GFP and anti-RFP antibodies, respectively. H⁺-ATPase was used as a PM marker. **(C)** Quantification of EFR protein abundance in total and PM fractions from **(B).** Band intensities were quantified using ImageJ and normalized to H⁺-ATPase. Different letters indicate statistically significant differences (one-way ANOVA with Tukey’s HSD, P < 0.05). **(D)** Pathogenicity assays of *S. flexneri* 5a and *Pst* in *Arabidopsis* PRR mutants. Bacterial growth was assessed in Col-0, *fls2*, and *efr* mutants at 3 dpi. Data represent mean ± SD. Different letters indicate statistically significant differences (one-way ANOVA with Tukey’s HSD, P < 0.05).


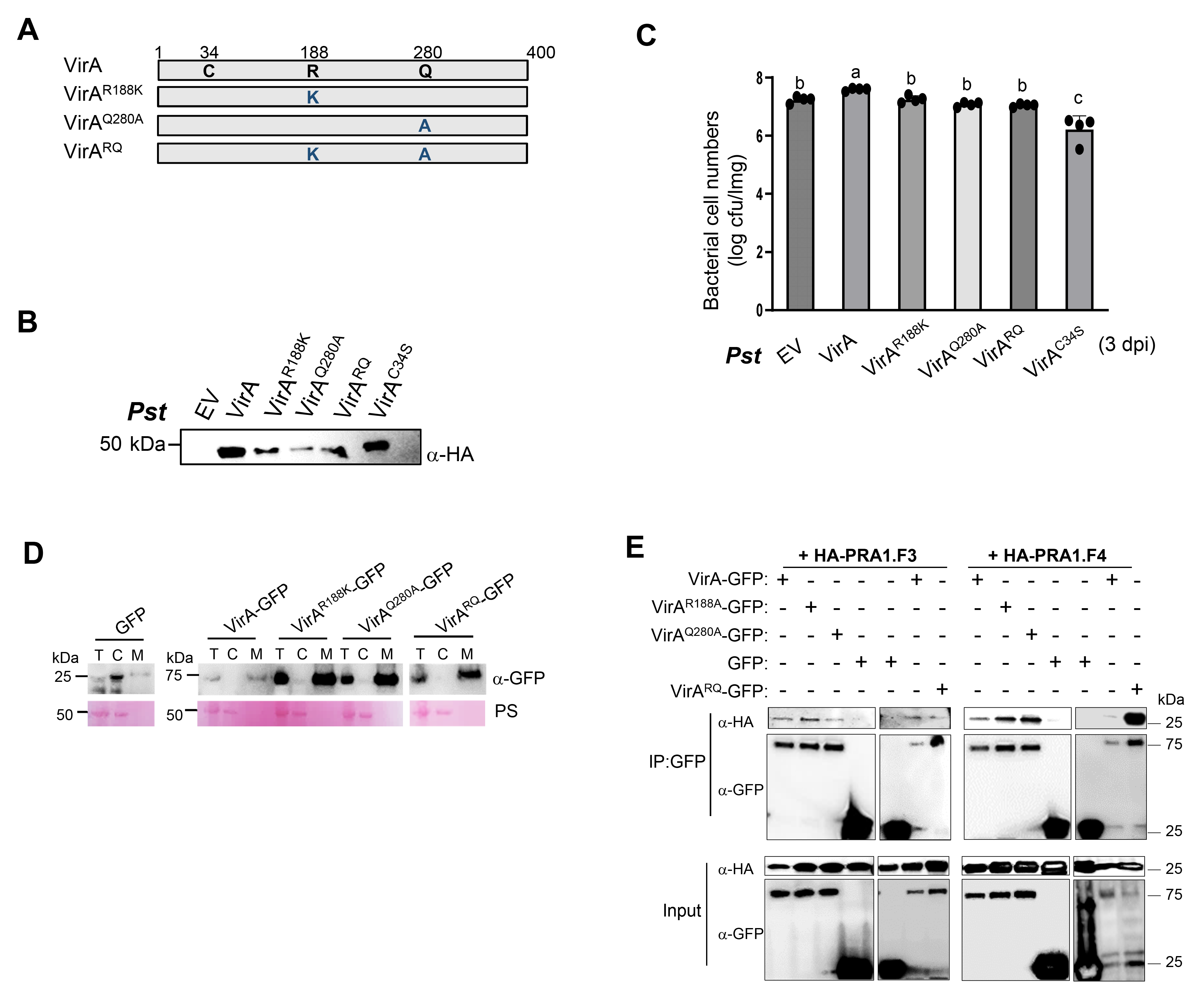


**Figure S10. TBC domain residues R188 and Q280 are critical for VirA-mediated virulence in plants. (A)** Schematic diagrams indicate the positions of site-directed mutations in VirA variants. **(B)** Detection of wild-type and mutant VirA proteins expressed in *Pst* by immunoblotting of total protein extracts using anti-HA antibody. **(C)** Bacterial growth in Arabidopsis seedlings flood-inoculated with *Pst* strains carrying an EV, VirA, VirA^R188K^, VirA^Q280A^, VirA^RQ^ (double mutant), or VirA^C34S^. Bacterial populations were quantified at 3 dpi. Data represent means ± SD (n = 4). Different letters indicate statistically significant differences as determined by a one-way ANOVA followed by Tukey’s HSD test (P < 0.05). **(D)** Membrane association of VirA is independent of its GAP activity. GFP-tagged WT VirA and the GAP-inactive mutants VirA^R188K^, VirA ^Q280A^, and VirA^RQ^ were expressed in *N. benthamiana* leaves, and total (T), cytosolic (C), and membrane (M) protein fractions were prepared at 2 dpi. Fractions were analyzed by immunoblotting using the indicated antibodies. **(E)** GAP activity is dispensable for PRA1 binding. Total protein extracts from *N. benthamiana* leaves expressing GFP-tagged VirA variants and HA-tagged PRA1 isoforms were subjected to co-IP using GFP-Trap agarose beads. Interacting proteins were detected by immunoblotting with anti-GFP and anti-HA antibodies.


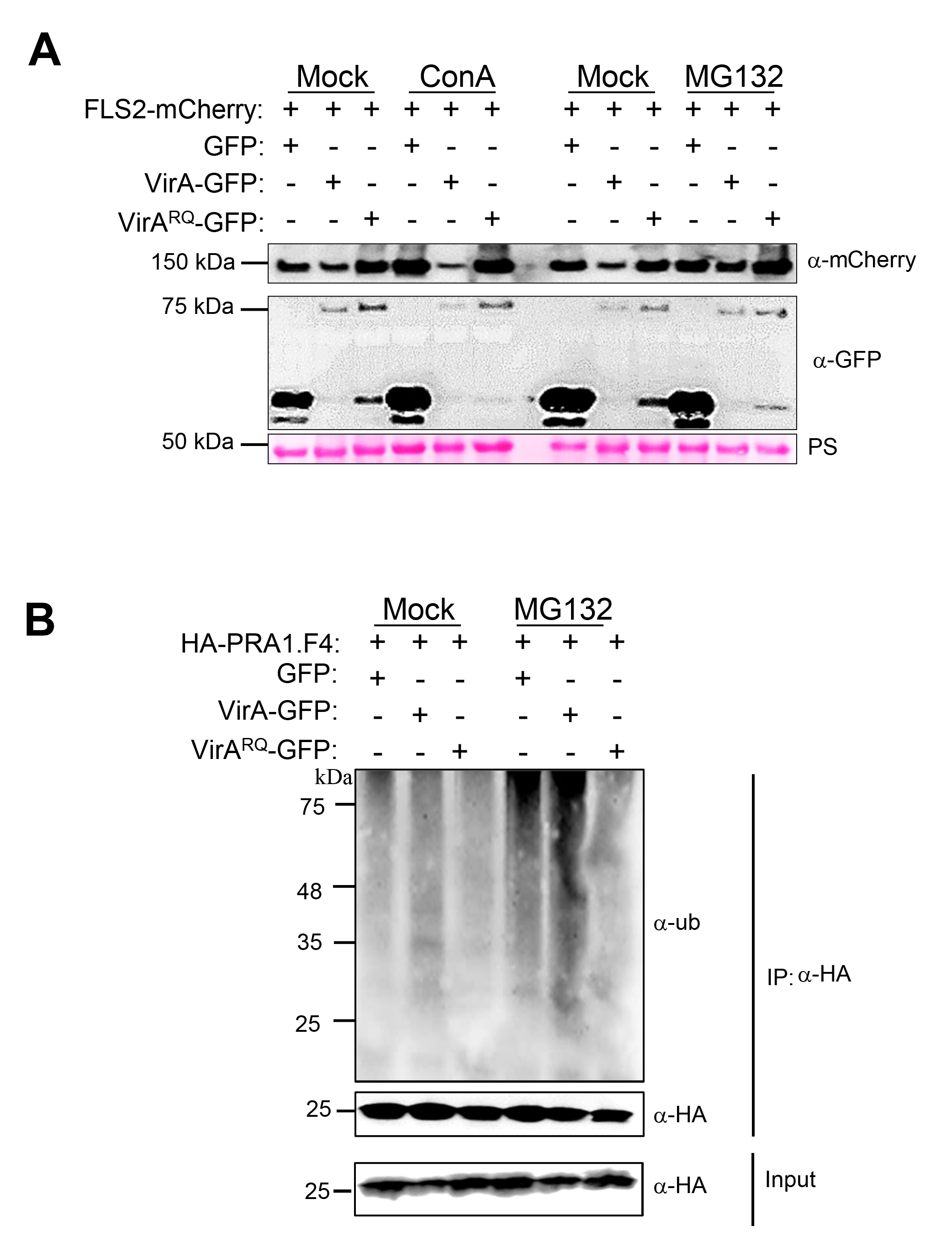


**Figure S11. VirA-dependent degradation of FLS2 is proteasome-dependent. (A)** VirA induces proteasome-dependent degradation of FLS2. Leaves co-expressing FLS2–mCherry and VirA–GFP were treated at 24 hpi with either 1 µM concanamycin A (autophagy inhibitor) or 50 µM MG132 (proteasome inhibitor). Total proteins were extracted 12 h later and analyzed by immunoblotting with the indicated antibodies **(B)** VirA-dependent ubiquitination of PRA1.F4 is weak in *N. benthamiana.* PRA1.F4 was co-expressed with VirA or VirA^RQ^ in *N. benthamiana* leaves via *Agrobacterium*-mediated infiltration. At 24 hpi, leaves were treated with 50 μM MG132 to block proteasomal degradation. At 12 h after MG132 treatment, total protein extracts were subjected to immunoprecipitation using anti-HA-conjugated magnetic beads. Ubiquitinated forms of PRA1.F4 were detected by immunoblotting with anti-ubiquitin antibody.


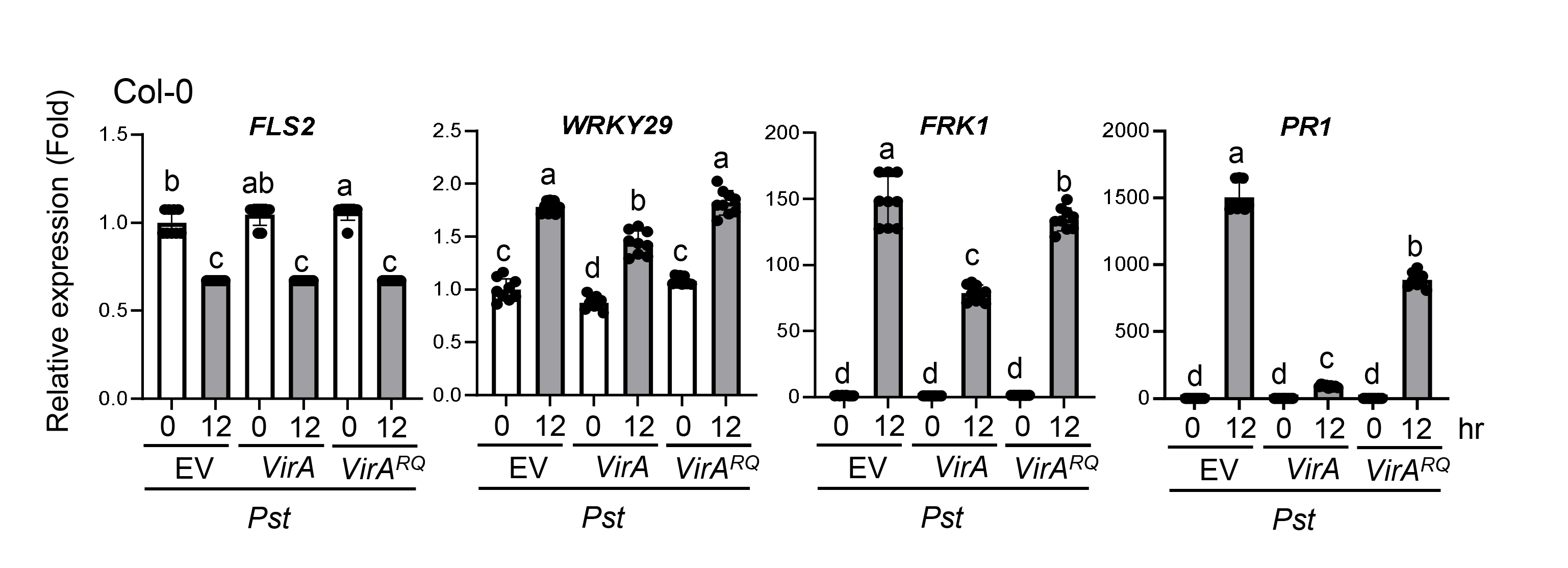


**Figure S12. VirA suppresses *FLS2*-dependent defense gene expression without altering *FLS2* transcript levels.** qRT–PCR analysis of *FLS2* and *FLS2* downstream defense marker genes (*WRKY29, FRK1*, and *PR1*) in Arabidopsis Col-0 plants following infection with *Pst* carrying empty vector (EV), VirA, or the GAP-deficient mutant VirA^RQ^. Leaf tissues were harvested at 0 and 12 h post infection (hpi). Gene expression levels were normalized to an internal reference gene (*Actin*) and are presented relative to the 0 h EV control. Bars represent mean ± SD from three independent biological replicates. Different letters indicate statistically significant differences (one-way ANOVA with Tukey’s HSD test, P < 0.05).


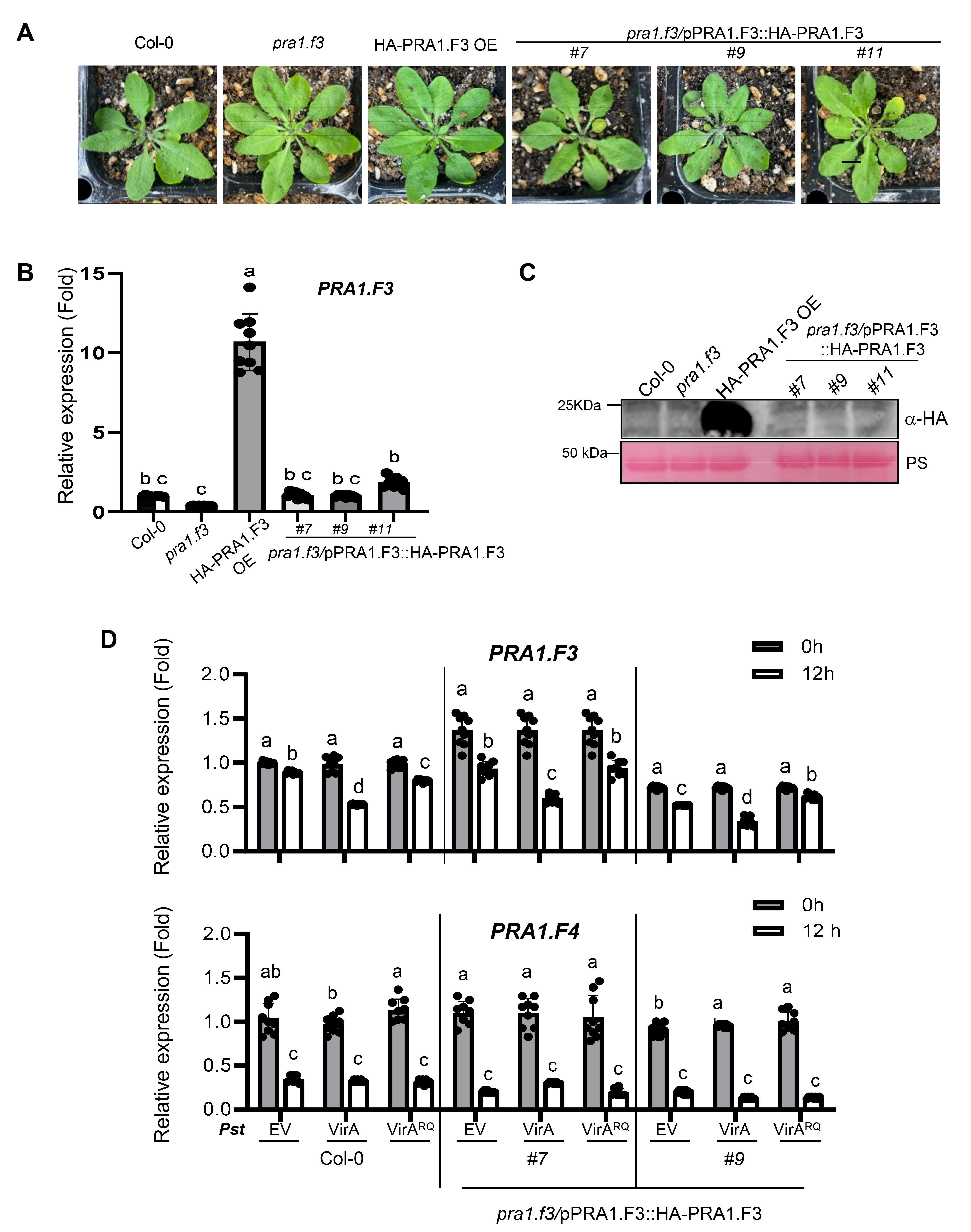


**Figure S13. Characterization of Arabidopsis *pra1.f3*/pPRA1.F3::HA-PRA1.F3 transgenic plants. (A**) Representative growth phenotypes of 4-week-old *pra1.f3*/pPRA1.F3::HA-PRA1.F3 transgenic plants, showing their normal development compared with wild-type Col-0. **(B)** Transcriptional expression of *PRA1.F3* in transgenic plants compared with that in Col-0 and *pra1.f3* mutant, as determined by qRT-PCR. Transcript levels were normalized to *AtActin*. Data represent means ± SD (n = 9). Different letters indicate statistically significant differences as determined by a two-way ANOVA followed by Tukey’s HSD test (P < 0.05). **(C)** Protein expression of HA-tagged PRA1.F3 in the transgenic plants. Total protein extracts were analyzed by immunoblotting using an anti-HA antibody. PS staining of RuBisCO served as a loading control. **(D)** VirA-dependent transcriptional repression of *PRA1.F3* during *Pst* infection. Col-0 and *pra1.f3*/pPRA1.F3::HA-PRA1.F3 transgenic lines were flood-inoculated with *Pst* strains harboring an EV, VirA, or VirA^RQ^ at 5 × 10⁵ cfu/ml. Transcript levels of *PRA1.F3* and *PRA1.F4* were measured at 6 and 12 hpi using qRT-PCR and normalized to *AtActin*. Data represent means ± SD (n = 9). Different letters indicate statistically significant differences as determined by a two-way ANOVA followed by Tukey’s HSD test (P < 0.05).


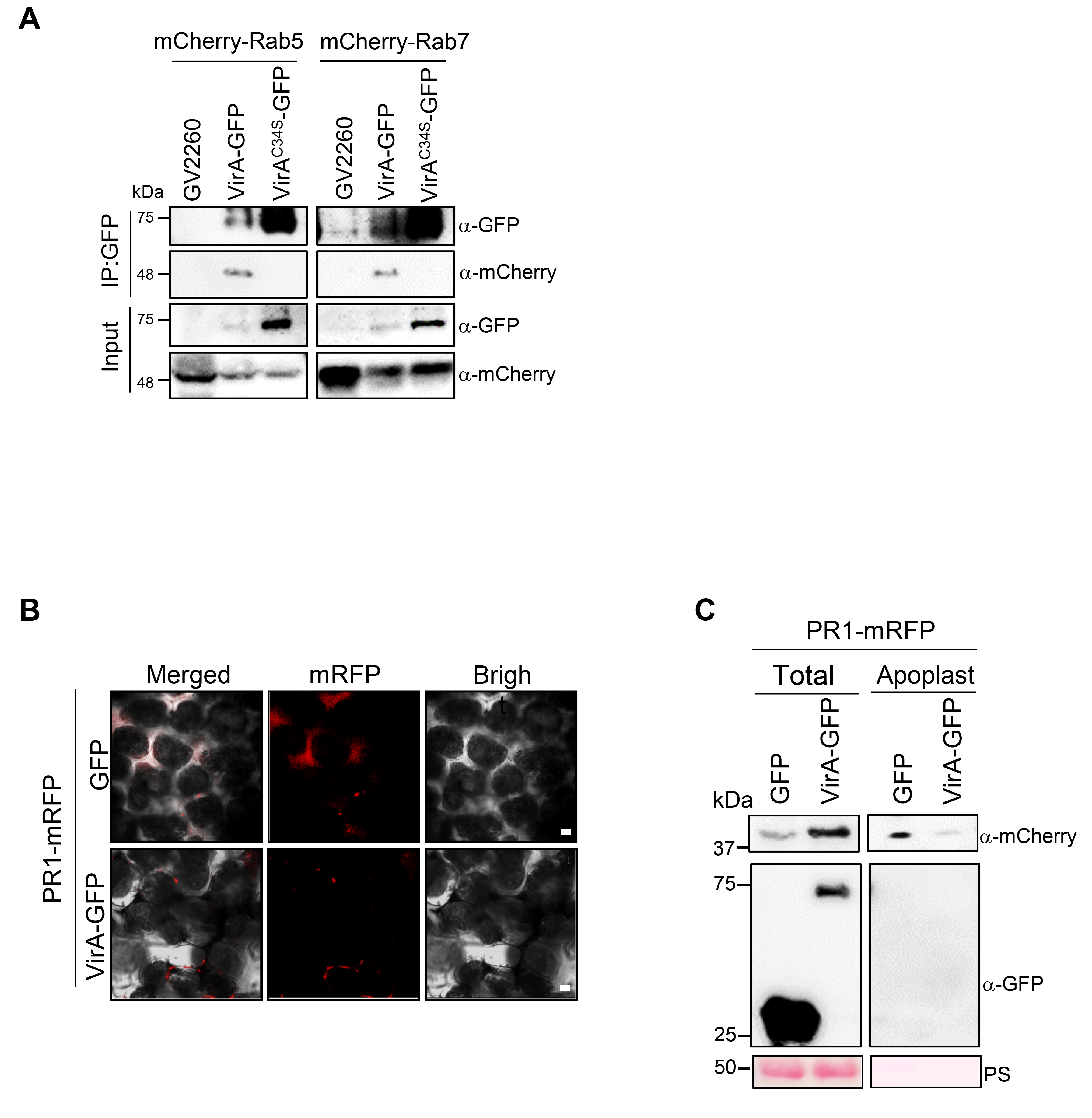


**Figure S14. VirA associates with Rab5 and Rab7 in plant cells.** VirA-GFP interacts with plant Rab proteins. VirA-GFP or VirA^C34S^-GFP was co-expressed with mCherry-Rab5 or mCherry-Rab7 in *N. benthamiana* leaves by *Agrobacterium-*mediated infiltration. Total protein extracts were prepared at 48 hpi and subjected to immunoprecipitation using anti-GFP antibody. Protein-protein interactions were analyzed by immunoblotting with anti-GFP and anti-mCherry antibodies.
